# Neanderthal coexistence with *Homo sapiens* in Europe was affected by herbivore carrying capacity

**DOI:** 10.1126/sciadv.adi4099

**Published:** 2023-09-22

**Authors:** Marco Vidal-Cordasco, Gabriele Terlato, David Ocio, Ana B. Marín-Arroyo

**Affiliations:** ^1^ Grupo I+D+i EvoAdapta (Evolución Humana y Adaptaciones durante la Prehistoria), Dpto. Ciencias Históricas, Universidad de Cantabria, Avd, Los Castros 44, 39005 Santander, Spain.; ^2^ Mott MacDonald Ltd., 22 Station Road, Cambridge, UK.

## Abstract

It has been proposed that climate change and the arrival of modern humans in Europe affected the disappearance of Neanderthals due to their impact on trophic resources; however, it has remained challenging to quantify the effect of these factors. By using Bayesian age models to derive the chronology of the European Middle to Upper Paleolithic transition, followed by a dynamic vegetation model that provides the Net Primary Productivity, and a macroecological model to compute herbivore abundance, we show that in continental regions where the ecosystem productivity was low or unstable, Neanderthals disappeared before or just after the arrival of *Homo sapiens*. In contrast, regions with high and stable productivity witnessed a prolonged coexistence between both species. The temporal overlap between Neanderthals and *H. sapiens* is significantly correlated with the carrying capacity of small- and medium-sized herbivores. These results suggest that herbivore abundance released the trophic pressure of the secondary consumers guild, which affected the coexistence likelihood between both human species.

## Teaser

Herbivore abundance influenced the spatiotemporal replacement patterns of Neanderthals by H. sapiens in Europe.

## INTRODUCTION

Building on the Malthusian population theory, Darwin stressed the evolutionary importance of interspecific competition: “We have reason to believe that species in a state of nature are limited in their ranges by competition of other organic beings quite as much as, or more than, by adaptation to particular climates” (*1*). This idea underpins a major concept in ecology: the competitive exclusion principle, which states that complete competitors cannot coexist over the long term (*2*). More recent studies revealed that although coexistence between competitor species is possible, it requires a complex balance in which resource availability and ecological niches determine species population dynamics and, therefore, their coexistence likelihood (*3*). Thus, ecological requirements and trophic resource availability are instrumental for species coexistence.

As *Homo sapiens* populations expanded out of Africa in successive waves and arrived in new environments, they were added to the secondary consumer guild. Following trophic cascade postulates, it has been proposed that *H. sapiens* increased the trophic pressure on herbivores and triggered the disappearance of some of their predators (*4*, *5*). However, the pace and extent of these extinctions were spatially and temporally diverse. In Europe, one of the first species that became extinct after the arrival of *H. sapiens* was *Homo neanderthalensis*. Rather than a rapid and straightforward replacement of Neanderthals by *H. sapiens*, the Middle to Upper Paleolithic transition (MUPT) was characterized by a mosaic of cultural and biological landscapes lasting several thousand years (*6*–*8*). Ancient DNA studies showed that our species and Neanderthals interbred (*9*) and, therefore, coexisted in some regions (*10*–*12*); nonetheless, in other areas of Europe, Neanderthals were quickly replaced by *H. sapiens* or even disappeared a few millennia before their arrival (*13*–*15*). The factors that triggered this spatiotemporal replacement pattern are unknown.

Greenland ice cores indicate that during the marine isotope stage 3 (MIS3) [ca. 60–30 thousand years (ka) before present (BP)], global climatic conditions experienced rapid and severe oscillations (i.e., Dansgaard-Oeschger events) between full glacial (stadial) and milder (interstadial) conditions (*16*). Nevertheless, the amplitude of these climatic fluctuations differed between European regions (*17*, *18*) and so did the oscillations in the plant and herbivore biomass (*19*, *20*). While some biogeographic regions served as refugia for human populations during the coldest periods of MIS3, other areas experienced harsher climatic conditions and discontinuities in the human settlement (*21*, *22*). A source-sink model has been proposed to explain these population processes of colonization, coexistence, fragmentation, isolation, and replacement. Populations would persist in high-quality habitats (“source areas”) but could not prevail in the long term in the low-quality ones (“sink areas”) (*23*). Neanderthals and *H. sapiens* had wide climatic tolerances (*24*, *25*) and exploited similar trophic niches (*26*–*28*). Therefore, it is plausible that they would have shared the same source and sink areas, with sink areas being prone to quicker species replacement and source areas allowing a longer persistence of both human species due to the abundance and stability of their trophic resources. However, this hypothesis has not been demonstrated to date.

This study tests whether the regional differences in the timing of the Neanderthal disappearance, the spread patterns of *H. sapiens*, and the temporal overlap between both human species were affected by the carrying capacity (CC) of the ecosystems that they inhabit in Europe (34°S to 55°N; −11°E to 30°W). The key goals of this study are (i) to quantify the ecosystem productivity during MIS3 in each European region, (ii) to provide an updated chronological frame of the MUPT, and (iii) to assess the association between the ecosystem productivity and the spatial and temporal replacement patterns of Neanderthals by *H. sapiens*. To this end, we first compiled an extensive dataset of chronometric dates and herbivore species recovered from archaeo-paleontological levels MIS3 dated. Then, we tested the validity of climate simulation obtained from an atmospheric general circulation model against different sets of paleoclimatic proxies (for details, see the “Paleoclimate reconstruction and validation” section in Materials and Methods). Bias-corrected paleoclimate values with the delta method were used as driver inputs into a process-based dynamic vegetation-terrestrial ecosystem model that estimated a continuous series of net primary productivity (NPP) between 55 and 30 ka BP in each archaeological and paleontological site. Then, we delimited the biogeographic regions of Europe according to their paleoclimatic conditions, the herbivore guild composition, and the temporal trends in NPP during MIS3. We used hierarchical Bayesian age models (BAMs) and optimal linear estimations (OLEs) to establish an updated chronology of the Neanderthals’ disappearance and their replacement by *H. sapiens* in each region. Last, we estimated the CC of each herbivore species in each region with a macroecological model validated with present-day observations across a broad range of terrestrial ecosystems. We carried out spatial regression analyses to assess the association between the ecosystem CC and the timing of the MUPT.

## RESULTS

### Regional differences in the ecosystem CC

**Fig. 1.**
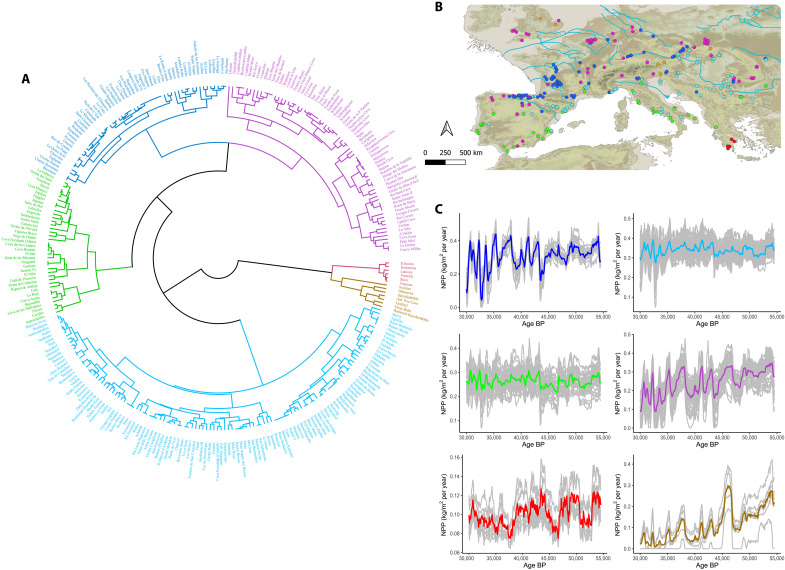
Net primary productivity. (**A**) Dendrogram clustering plot according to the NPP differences obtained from the dCORT dissimilarity index. Each color indicates one cluster. (**B**) Archaeo-paleontological sites where NPP was estimated. Colors correspond with the same colors used in the dendrogram plot. (**C**) Temporal evolution of NPP in each archaeo-paleontological site grouped according to the clusters obtained from the dCORT index. The colored line represents the mean, corresponding with the color used in (A) and (B).

**Fig. 2.**
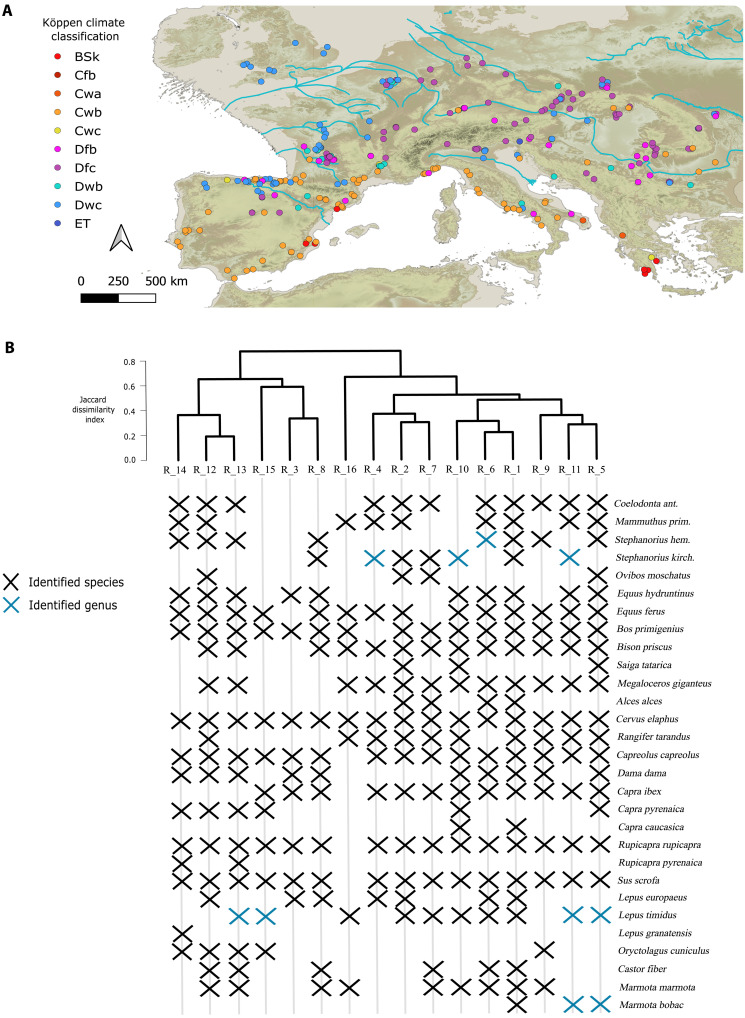
Climate and herbivore species during MIS3. (**A**) Köppen-Geiger climate classification in each archaeo-paleontological site included in this study. D, continental; C, temperate; E, polar; T, tundra; w, dry winter; f, no dry season; s, dry summer; a, hot summer; b, warm summer; c, cold summer. All climate variables were obtained from the work of Armstrong *et al.* (*90*) after bias correction (see the “Paleoclimate reconstruction and validation” section for details). (**B**) Cluster dendrogram with the classification of each herbivore paleocommunity in each region according to the Jaccard Dissimilarity Index (JDI). The figure depicts the herbivore species recovered in each region during MIS3.

**Fig. 3.**
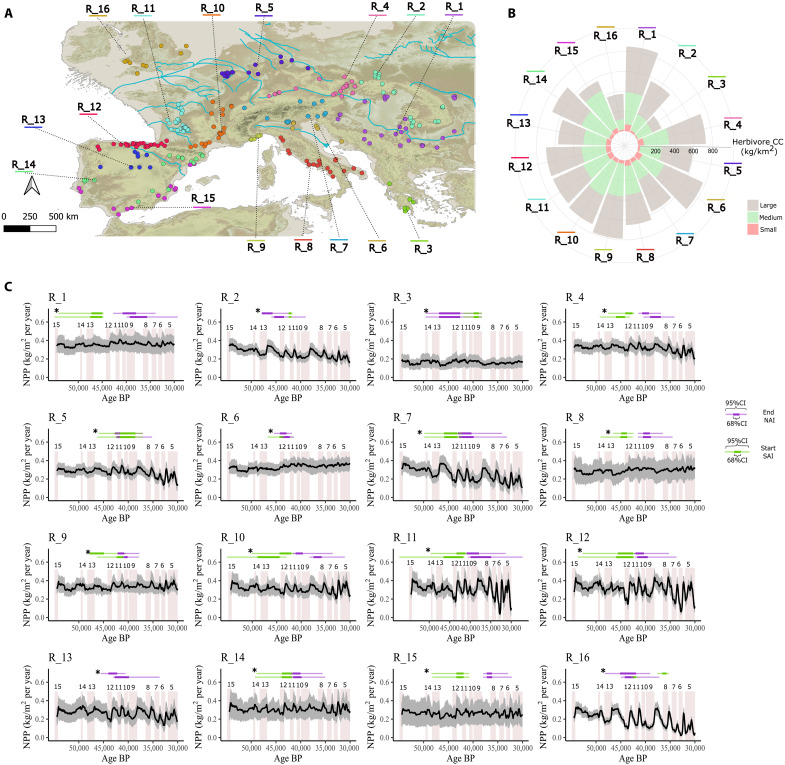
Biogeographic regions of Europe. Biogeographic regions during MIS3 according to the climate conditions, the NPP, and the herbivore guild composition. (**A**) Dots represent the archaeo-paleontological sites included in the study. (**B**) Mean CC (kg/km^2^ per year) of large-sized (>300 kg), medium-sized (20 to 300 kg), and small-sized (<20 kg) herbivores in each region during MIS3. (**C**) Temporal evolution of the NPP during MIS3 in each biogeographic region. Vertical numbered bars represent the stadial phases. Horizontal bars show the timing of the NAI (in purple) disappearance at 68% CI (with bold, thicker bar) and 95% CI, and the timing of the SAI (in green) appearance at 68% and 95% CI. Asterisks (*) indicate that the BAMs were run with the most restrictive filtering criteria (filtering criteria 2; for details, see the “Chronological models” section in Materials and Methods).

According to the temporal trends of NPP (Fig. 1), the paleoclimate conditions and the herbivore guild composition (Fig. 2) during MIS3, 16 biogeographic regions across Europe were identified (for details, see the “Biogeographic regions” section in Supplementary Text; Fig. 3). In Eastern Europe (R_1, R_2, and R_3), the highest and more constant NPP is observed along the lower Danube Basin (R_1), which is proposed as one of the first entering routes of modern humans (Table 1) (*29*). Conversely, the sharpest fluctuations in NPP are observed in the northern sites around the Carpathians (R_2), and the lowest NPP are observed in the southerly Mediterranean sites from the Balkan peninsula (R_3). Consequently, the highest CC of all-sized herbivores is observed around the lower Danube Basin (R_1), followed by the Carpathian (R_2) and the Balkan Mediterranean regions (R_3). Likewise, in Central Europe (R_4 and R_5), NPP and CC of all-sized herbivores were higher in the upper Danube Basin (R_4) than in the northern (latitude, ≥50°N) sites (R_5; Table 1).

**Table 1.** Summary statistics of the ecosystems’ productivity. The mean value, SD, and coefficient of variation (CV) of the NPP in each biogeographic region during MIS3. The mean value, lower limit (LL), and upper limit (UL) at 95% CI for the estimated CC of small-sized (1 to 20 kg), medium-sized (20 to 300 kg) and large-sized (>300 kg) herbivores during MIS3. Each value was classified (C) on the basis of its percentile as follows: low (L) for values <0.25 percentile, medium (M) for values between the 0.25 and 0.75 percentiles, and high (H) for values >0.75 percentile. *Region names are broad designations to aid readers in geographically locating them.

Region*	NPP (kg/m^2^ per year)					Small (kg/km^2^ per year)				Medium (kg/km^2^ per year)				Large (kg/km^2^ per year)			
	Mean	C	SD	CV	C	Mean	C	95% CI		Mean	C	95% CI		Mean	C	95% CI	
								LL	UL			LL	UL			LL	UL
Lower Danube Basin (R_1)	0.357	H	0.02	5.13	L	65.39	H	50.23	85.14	321.66	H	247.06	418.78	462.86	H	355.51	602.62
Carpathian region (R_2)	0.262	L	0.05	17.74	H	20.29	M	16.2	25.41	176.3	M	140.76	220.83	363.6	H	290.29	455.42
Balkan Mediterranean region (R_3)	0.162	L	0.02	10.38	M	11.16	L	8.87	14.04	168.28	L	133.77	211.7	106.24	L	84.45	133.65
Upper Danube Basin (R_4)	0.316	H	0.03	10.52	M	59.73	H	46.73	76.35	271.66	M	212.53	347.23	396.5	H	310.2	506.81
Northern Europe (R_5)	0.273	M	0.04	15.17	M	21.24	M	16.91	26.7	231.79	M	184.46	291.27	337.61	H	268.68	424.24
Adriatic region (R_6)	0.331	H	0.02	7.22	L	62.08	H	48.26	79.86	256.32	M	199.26	329.73	457.02	H	355.27	587.9
Prealpine region (R_7)	0.268	M	0.06	22.63	H	40.34	M	32.15	50.61	186.82	M	148.91	234.39	348.95	H	278.14	437.8
Italian Mediterranean region (R_8)	0.292	M	0.02	7.69	L	54.64	H	43.18	69.14	289.4	M	228.69	366.21	306.08	M	241.88	387.32
Ligurian region (R_9)	0.334	H	0.02	7.45	L	32.54	M	25.25	41.92	327.7	H	254.33	422.22	427.03	H	331.43	550.22
Rhone basin (R_10)	0.312	H	0.04	11.73	M	37.63	M	29.5	48.01	364.79	H	285.96	465.34	310.35	H	243.29	395.9
Aquitaine region (R_11)	0.304	M	0.07	23.61	H	53.77	H	42.28	68.39	278.69	M	219.14	354.43	357.02	H	280.72	454.04
Cantabrian region (R_12)	0.306	M	0.07	23.25	H	51.21	H	40.24	65.18	260.3	M	204.52	331.28	383.7	H	301.48	488.34
Northern Meseta (R_13)	0.264	M	0.04	17.02	M	50.92	H	40.63	63.81	241.63	M	192.8	302.83	273.34	M	218.11	342.57
Iberian Medomediterranean region (R_14)	0.307	H	0.03	9.24	L	31.25	M	24.54	39.8	321.25	H	252.26	409.1	347.16	H	272.62	442.1
Iberian Mediterranean region (R_15)	0.263	L	0.02	9.02	L	48.4	H	38.63	60.63	405.91	H	323.99	508.53	108.29	M	86.44	135.67
Britain (R_16)	0.196	L	0.08	41.7	H	13.46	L	10.83	16.73	103.88	L	83.57	129.13	254.56	M	204.78	316.45

In Western Europe (from R_6 to R_16), the highest NPP mean is observed in mid-latitude Mediterranean regions (R_6, R_9, R_10, and R_14), followed by the Atlantic areas of the Aquitaine (R_11) and Cantabrian (R_12) regions, whereas the lowest NPP is observed in the northernmost (R_16) and southernmost (R_15) regions because low temperatures and precipitations were a constraining factor (Fig. 3). In Western Europe, the sites from Britain (R_16), northern Iberia (R_12), west France (R_11), and the Prealps (R_7) show the highest coefficient of variation in the estimated NPP (Table 1) and, therefore, experienced the sharpest fluctuations in plant biomass. The coefficient of variation of NPP in the Prealps (R_7) is three times larger than in the remaining regions of the Italian peninsula (Table 1). Likewise, in the Northern Meseta of Iberia (R_13), the coefficient of variation of NPP is twice as large as in the Mediterranean regions from the Iberian Peninsula (Table 1). Therefore, the stadial-interstadial transitions had a meaningful effect on the plant biomass in mid- and high-altitude areas. Those regions from Western Europe with the lowest NPP (R_15 and R_16) had the lowest CC of large-sized herbivores and vice versa (R_6, R_9, and R_10). Moreover, the highest CC of small or medium-sized herbivores is observed in regions with high NPP (R_6, R_9, R_10, and R_14; Table 1) or with lower biomass of large-sized herbivores (R_8 and R_15; Fig. 2). Accordingly, differences in the NPP and the herbivore guild composition between regions led to remarkable differences in the herbivore CC during MIS3.

### Chronology of the MUPT in Europe

The filtering criteria used to select the chronometric determinations incorporated into the BAMs affected the outcomes obtained for the timing of the MUPT in some regions. When only chronometric dates obtained from materials with signs of human manipulation (e.g., bones with anthropic marks or charcoals from hearths) are used [ filtering criteria 2 (FC 2)], the end of the Neanderthal-affiliated industries (NAIs) is systematically older and the beginning of the *H. sapiens*–affiliated industries (SAIs) is younger (Tables 2 and 3). Likewise, when FC 2 is used, some techno-complexes are no longer represented (e.g., SAI in the Adriatic region, R_6; Table 3). Thus, whereas the most restrictive filtering criteria produce more robust results, they also generate a smaller sample size, eliminating the representation of some cultures in some regions. All analyses in this study were performed with both filtering criteria (FC 1 and FC 2) to estimate the sensitivity of the conclusions to the choice of chronometric dates.

**Table 2.** Chronology with filtering criteria 1. Highest mode of the posterior density (age) for the timing of the NAI disappearance and the SAI appearance in each region according to the first (FC1) chronometric filtering criteria (for details, see the “Chronological models” section in Materials and Methods). Highest probability density (HPD) at 68% and 95% CI for each chronology estimate. Chronologies are expressed in ka BP.

Region	NAI disappearance					SAI appearance				
	Age	HPD at 68% CI		HPD at 95% CI		Age	HPD at 68% CI		HPD at 95% CI	
Lower Danube Basin (R_1)	37.79	35.65	39.25	29.28	39.94	45.73	44.99	47.48	44.75	54.92
Carpathian region (R_2)	44.54	43.36	45.36	38.97	45.93	42.16	42.00	42.30	41.64	42.58
Balkan Mediterranean region (R_3)	44.85	42.62	46.87	38.25	49.60	39.28	38.85	39.85	38.15	41.95
Upper Danube Basin (R_4)	38.02	36.85	39.10	34.01	40.40	45.02	44.09	46.07	42.97	49.33
Northern Europe (R_5)	41.75	40.50	42.51	35.13	43.07	39.93	38.49	41.74	37.02	46.42
Adriatic region (R_6)	42.88	42.22	43.43	41.33	44.06	42.98	42.26	44.31	41.46	46.66
Prealpine region (R_7)	41.86	39.87	42.82	33.14	43.21	44.30	43.14	45.97	42.08	49.99
Italian Mediterranean region (R_8)	39.68	38.93	40.51	34.51	41.51	44.1	43.57	45.21	42.09	48.77
Ligurian region (R_9)	40.48	40.11	40.84	37.85	41.24	41.45	40.91	42.33	40.59	46.30
Rhone basin (R_10)	36.74	35.65	37.59	30.68	38.45	46.23	44.42	48.94	43.02	55.17
Aquitaine region (R_11)	38.26	34.81	39.99	27.43	40.85	43.03	41.69	46.49	41.40	57.27
Cantabrian region (R_12)	40.78	39.31	41.74	33.57	42.47	43.67	42.37	46.24	41.53	53.85
Northern Meseta (R_13)	42.09	39.87	42.75	33.67	43.06	–	–	–	–	–
Iberian Medomediterranean region (R_14)	40.88	39.82	41.51	35.07	41.86	42.53	41.76	43.86	41.48	49.23
Iberian Mediterranean region (R_15)	36.65	36.13	37.21	32.15	37.98	42.63	41.92	43.38	40.71	48.23
Britain (R_16)	43.31	41.93	44.19	37.21	45.20	42.16	41.94	42.41	41.48	42.88

**Table 3.** Chronology with filtering criteria 2. Highest mode of the posterior density (age) for the timing of the NAI disappearance and the SAI appearance in each region according to the chronometric filtering criteria 2 (for details, see the “Chronological models” section in Materials and Methods). HPD at 68% and 95% CI for each chronology estimate. Chronologies are expressed in ka BP.

Region	NAI disappearance					SAI appearance				
	Age	HPD at 68% CI		HPD at 95% CI		Age	HPD at 68% CI		HPD at 95% CI	
Lower Danube Basin (R_1)	39.54	37.94	40.75	33.88	42.72	45.68	44.95	47.24	44.73	54.07
Carpathian region (R_2)	45.93	45.68	45.50	47.20	48.86	42.15	41.87	42.40	41.63	42.61
Balkan Mediterranean region (R_3)	44.85	42.62	46.87	38.25	49.60	39.28	38.85	39.85	38.15	41.95
Upper Danube Basin (R_4)	39.97	39.20	40.70	36.74	41.47	43.29	42.55	44.24	41.79	47.67
Northern Europe (R_5)	42.22	41.05	42.81	36.94	43.29	39.98	38.47	41.72	37.08	46.21
Adriatic region (R_6)	43.40	42.85	44.19	41.76	45.39	–	–	–	–	–
Prealpine region (R_7)	42.19	40.33	43.07	34.12	43.41	44.35	43.16	45.97	42.09	49.97
Italian Mediterranean region (R_8)	39.72	39.12	40.45	36.49	41.51	44.1	43.57	45.21	42.09	48.77
Ligurian region (R_9)	40.49	40.82	42.15	37.75	41.25	41.37	44.92	47.82	40.43	45.90
Rhone basin (R_10)	40.53	39.47	41.33	33.44	42.01	42.97	42.00	44.44	41.31	50.14
Aquitaine region (R_11)	39.59	37.29	40.83	30.36	30.36	41.87	41.15	43.35	40.83	49.58
Cantabrian region (R_12)	41.32	40.33	42.02	35.28	42.35	43.53	42.33	42.33	41.53	52.92
Northern Meseta (R_13)	42.92	42.23	44.02	40.54	45.54	–	–	–	–	–
Iberian Medomediterranean region (R_14)	40.98	40.03	41.67	35.56	42.02	42.52	41.75	43.78	41.43	48.90
Iberian Mediterranean region (R_15)	36.66	36.18	37.23	32.92	37.99	42.63	41.93	43.38	40.75	48.41
Britain (R_16)	43.39	41.90	45.15	39.02	48.24	36.16	35.77	36.60	35.28	37.61

Because of the wide 95% confidence intervals (CIs) obtained from the BAMs, the end of the NAI and the beginning of the SAI cannot be associated with a specific stadial or interstadial phase in the lower Danube Basin (R_1). However, in R_1, the onset of the SAI was previous to the Greenland stadial (GS) 12 and the end of the NAI posterior to the GS-11, revealing a temporal overlap of, at least, 4.8 ka between both human species. On the contrary, in the Carpathian region (R_2) and the southern area of the Balkan Peninsula (R_3), the NAI disappearance predates the appearance of the SAI. Thus, in Eastern Europe, the NAI disappeared ~6 ka later in the lower Danube Basin (R_1) than in the Carpathian (R_2) and Balkan Mediterranean (R_3) regions (Fig. 3). Conversely, the SAI appeared at least 3.5 ka earlier in R_1 than in R_2 and R_3 (Fig. 3). Therefore, the NAI disappeared first in those regions where the SAI arrival took longer (R_2 and R_3) and, later, in the region with the earliest SAI appearance (i.e., R_1). These spatiotemporal replacement patterns remain consistent regardless of the filtering criteria used (FC 1 and FC 2).

In the upper Danube Basin (R_4), there was a temporal overlap of at least 3.3 ka between both techno-complexes. On the contrary, there was hardly any temporal overlap in Northern Europe (R_5; Fig. 3). Moreover, the NAI disappeared earlier in R_5 than in R_4 (Tables 2 and 3). Conversely, the SAI appeared earlier in R_4 than in R_5. In the Italian peninsula, the Mousterian disappeared first in the Adriatic area (R_6), followed by the Prealpine (R_7) and the Mediterranean (R_8) regions (Tables 2 and 3). In contrast, the SAI appeared first in the Mediterranean region (R_8), followed by the Adriatic (R_6) and the Prealpine (R_7) regions. This means that similar to Eastern and Central Europe, in the Italian peninsula, the SAI appeared first in the same regions with a longer Mousterian persistence and later in those regions with an earlier Mousterian disappearance (Fig. 3). However, unlike Eastern and Central Europe, in the Italian peninsula, there is a low difference (~1.1 ka) in the timing of the SAI appearance between regions, which suggests a rapid spread of modern humans.

In the French regions, the NAI disappeared after the arrival of the SAI, pointing toward a temporal overlap between the NAI and the SAI techno-complexes. On the other hand, the NAI ended between 1.5 and 2.6 ka earlier around the Ligurian (R_9) and the Rhône basin (R_10) than in the Aquitaine region (R_11), and the SAI appeared between 1.1 and 3.1 ka earlier in R_9 and R_10 than in R_11 (Tables 2 and 3). In the Iberian Peninsula, the Mousterian lasted at least 4.1 ka longer in the Mediterranean region of R_15 than in the Cantabrian region (R_12) and Northern Meseta (R_13). On the other hand, the SAI appeared first in the R_12 region, followed by the R_15 and the R_14 regions (Tables 2 and 3). Regardless of the chronometric filtering criteria used in the BAMs, the Mousterian in Britain (R_16) predates the SAI arrival, so the NAI and SAI did not overlap.

**Fig. 4.**
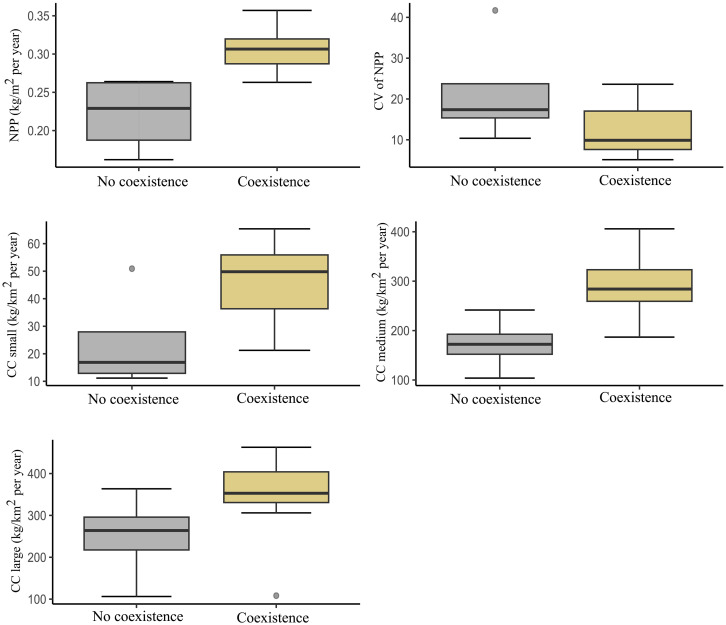
Ecosystem productivity and coexistence between Neanderthals and *H. sapiens*. Box and whisker plots showing the NPP, its coefficient of variation (CV), and the CC of small-, medium-, and large-sized herbivores in those regions with (Coexistence) and without (No coexistence) temporal overlap between Neanderthals and *H. sapiens*. The center line of each plot corresponds to the median. The box edges correspond to the first and third quartiles. The whiskers correspond to 1.5× the interquartile range, and the dots represent outliers.

Although the end of the NAI and the beginning of the SAI seem to be associated with specific NPP drops in several regions (R_2, R_4, R_5, R_7, R_10, R_11, and R_12; Fig. 3), two factors hinder us from establishing a direct link: first, the wide CIs derived from the chronological models (Tables 2 and 3) and, second, the sharp and quick fluctuations in NPP observed in these regions (Fig. 3). Thus, the end of the NAI and the beginning of the SAI lasted several stadial/interstadial phases in most regions and, therefore, several ups and downs in NPP. Nevertheless, the remarkable differences in the timing of the MUPT between regions shed light on the spatiotemporal patterns of the Neanderthal replacement by *H. sapiens* across Europe. In this connection, kernel density estimates (KDEs) suggest coexistence between NAI and SAI in the aforementioned overlapping areas identified by the BAM and OLE (movie S1), supporting the identified spatiotemporal replacement patterns. In 25% of the European regions, the end of the NAI predates the appearance of the SAI (R_2, R_3, R_13, and R_16). In these regions, NPP was, on average, 27% lower, and the coefficient of variation is 39% higher than in those regions with a temporal overlap between both human species (Table 1). Likewise, in those regions without coexistence between both human species, the CC of small-sized, medium-sized, and large herbivores was, on average, 49, 41, and 29% lower, respectively (Fig. 4). In turn, regions with more constant NPP (lower coefficient of variation) or higher CC of small- and medium-sized herbivores tended to witness a more prolonged coexistence between both species (Fig. 4).

### Ecological factors on the human replacement patterns

**Fig. 5.**
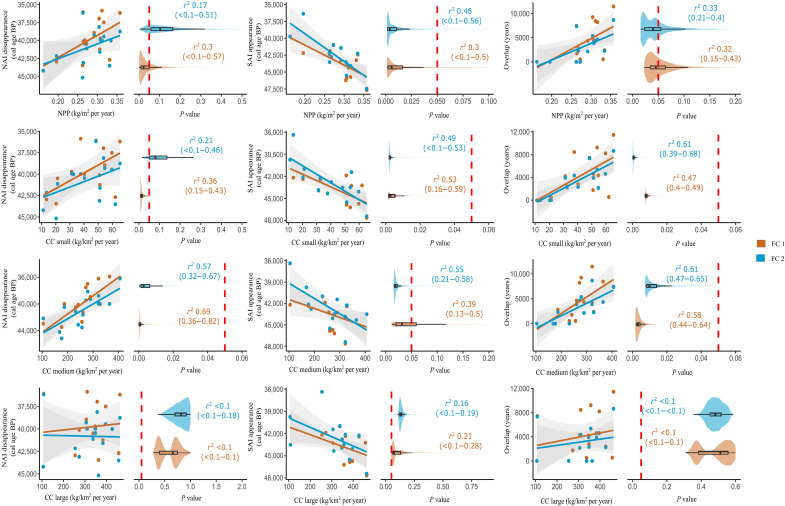
Correlation between dependent and independent variables. Dependent variables are as follows: the timing of the NAI disappearance, the SAI appearance, and the temporal overlap between the NAI and the SAI in each region. Independent variables are NPP and CC of small-, medium-, and large-sized herbivores. Each ESF test was performed with the chronometric filtering criteria that produced the FC 1 and 2 (for details, see “Chronological models” in Materials and Methods). Violin and box plots show the range of *P* values after the resampling procedure that incorporates the uncertainty at 95% CI of each dependent and independent variable. The vertical dashed red line indicates the threshold of 0.05 in the *P* value, so values to the left (*P* ≤ 0.05) are statistically significant.

NPP and CC of small-sized herbivores show a linear positive correlation with the timing of the NAI disappearance (the higher the NPP and CC, the later the NAI disappear), but these correlations are not statistically significant (*P* > 0.05; Fig. 5). The wide range of *P* values obtained after the permutation tests suggests that the associations between NPP, CC of small-sized herbivores, and the end of the NAI are sensitive to the chronometric filtering criteria used to reconstruct the chronology of the NAI disappearance. Likewise, CC of large-sized herbivores is uncorrelated with the end of the NAI in Europe. On the contrary, CC of medium-sized herbivores is significantly correlated (*P* < 0.05) with the timing of the NAI disappearance, and therefore, the techno-complexes associated with *H. neanderthalensis* persisted longer in those regions with a higher CC of medium-sized herbivores. This correlation remains significant after the bootstrap tests and is independent of the filtering criteria used to reconstruct the chronology of the NAI disappearance. According to the correlation coefficients obtained, between 32 and 82% of the variance in the timing of the NAI disappearance in Europe is explained by the CC of medium-sized herbivores (Fig. 5).

The onset of the SAI is negatively correlated with NPP and CC of small- and medium-sized herbivores (Fig. 5). Hence, the techno-complexes associated with *H. sapiens* appeared first in regions with a higher biomass of plants and a higher CC of small- and medium-sized herbivores. CC of large-sized herbivores is the only variable uncorrelated with the timing of the SAI appearance. However, the wide range of *P* values obtained after the permutation tests suggests that the significance level of the correlation between CC of medium-sized herbivores and the arrival of the SAI is sensitive to the chronometric filtering criteria. The remaining correlations are statistically significant independently of the two sets of chronometric filtering criteria (FC 1 and FC 2) and the uncertainty in the dependent and independent variables (Fig. 5).

NPP and CC of large-sized herbivores did not affect the temporal overlap between the NAI and SAI, which can be attributed to the reduced trophic pressure that is often exerted on large herbivores (further addressed in Discussion). However, CC of small- and medium-sized herbivores is significantly correlated with the temporal overlap between Neanderthals and *H. sapiens*. These correlations remain statistically significant independently of the chronometric filtering criteria and the permutation tests (Fig. 5). Therefore, results obtained suggest that the temporal overlap between both human species was significantly longer in those regions with a higher CC of small- and medium-sized herbivores. These results support the hypothesis that the observed replacement and overlap patterns between Neanderthals and *H. sapiens* during the MUPT were largely driven by differences in the herbivore CC between European regions.

### Sensitivity tests

**Fig. 6.**
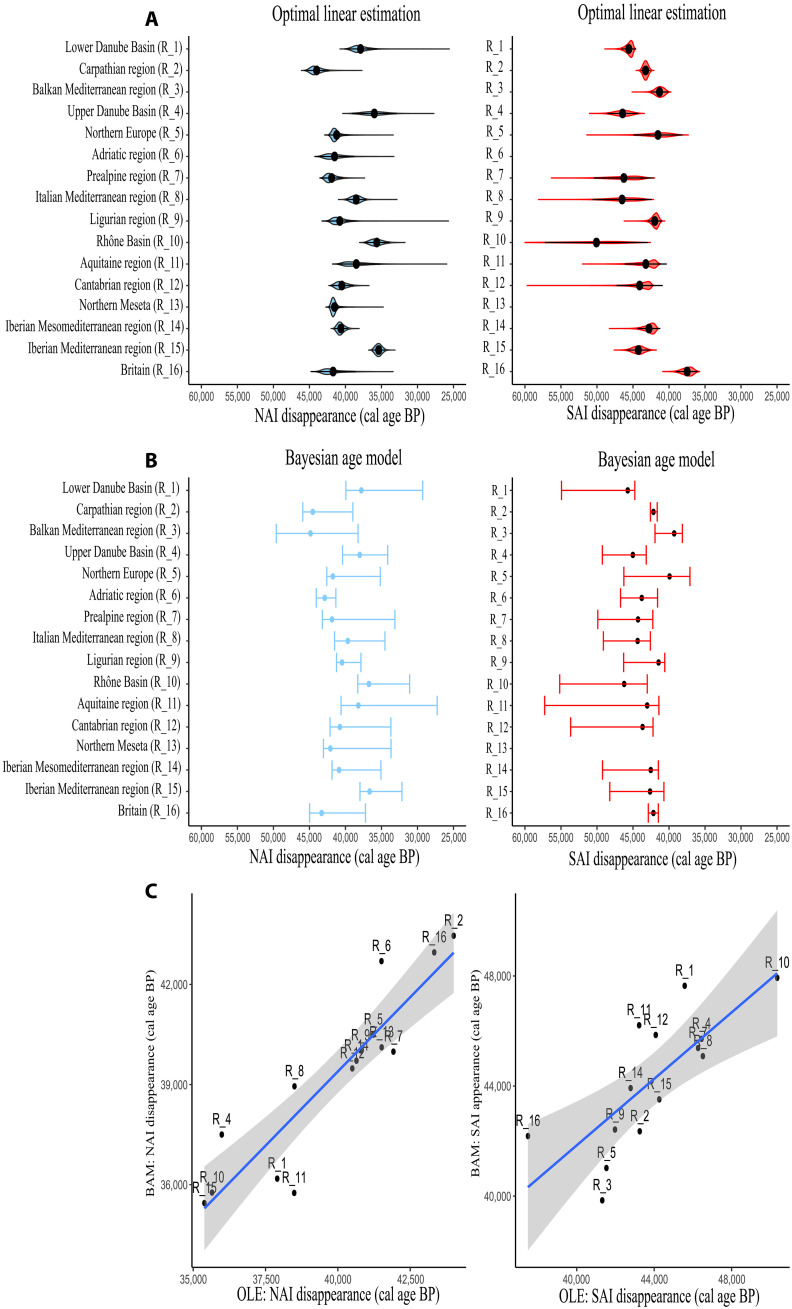
Comparison between OLE and BAM outputs. (**A**) Violin plot with the mean and the 95% CI for the end of the NAI (blue color) and the beginning of the SAI (red color) in each region according to OLEs. (**B**) Mean and 95% CI interval for the end of the NAI (in blue color) and the start of the SAI (red color) according to the BAMs. (**C**) Linear correlation between the chronologies obtained with BAM and OLE models. Each point represents one biogeographic region identified with the label code.

Results reported so far were based on the chronological estimates obtained from BAMs. We rerun the main analyses using OLEs to assess whether these results depend on the modeling approach. There is an overall agreement in the timing of the MUPT between OLE and BAM (Fig. 6), which validates the chronological aspects of this study. Notably, the effects of the herbivore CC on the timing of the NAI disappearance and the temporal overlap between NAI and SAI techno-complexes are also observed with the chronology obtained from OLE (fig. S1). Besides, the intercepts and slopes of these correlations do not differ when the correlation tests are performed with the chronological estimates obtained from the OLE model or with those obtained from the BAM models (table S1). Therefore, the results obtained in this study are not biased by the chronological modeling approach.

**Fig. 7.**
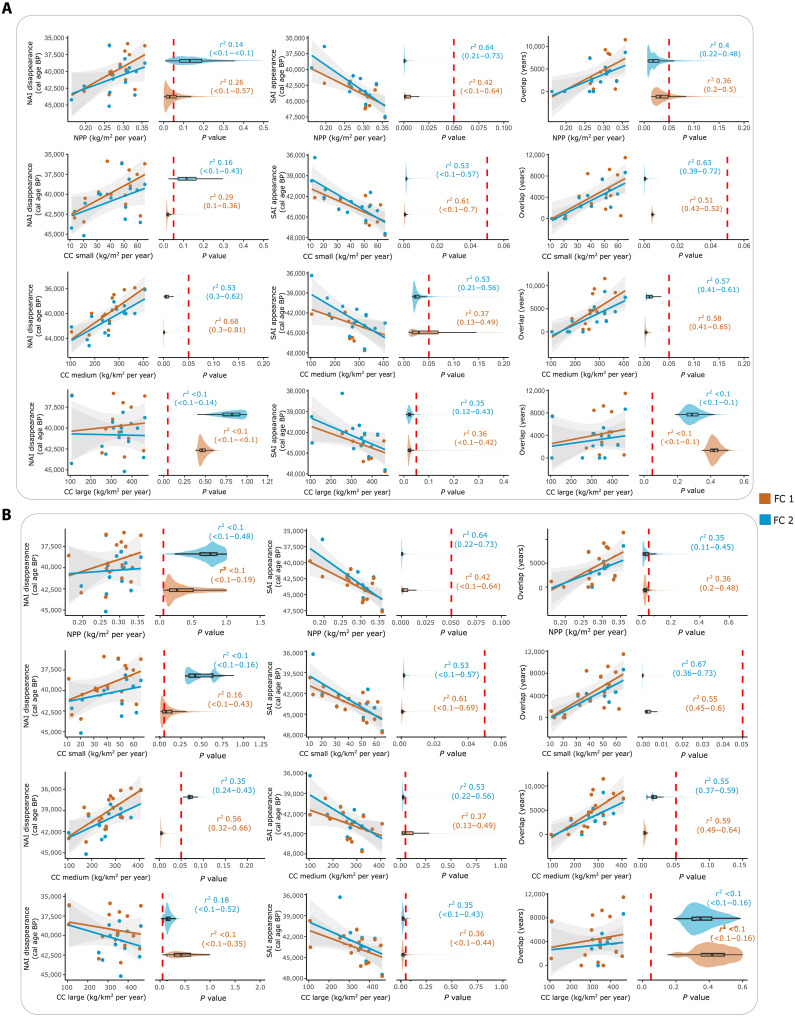
Sensitivity to the culture-species association uncertainty. Correlation between dependent and independent variables when (**A**) the Châtelperronian is excluded from the NAI and the Uluzzian is excluded from the SAI and (**B**) the Uluzzian is incorporated into the NAI. Each ESF test was performed with the chronometric FC 1 and 2. Violin and box plots show the range of *P* values after the resampling procedure that incorporates the uncertainty at 95% CI of each dependent and independent variable. The vertical dashed red line indicates the threshold of 0.05 in the *P* value, so values to the left (*P* < 0.05) are statistically significant.

An additional sensitivity test was carried out to assess to what extent the main results could be affected by incorporating the Châtelperronian and Uluzzian techno-complexes into the analyses. If these techno-complexes were removed, then the main results of this study would remain almost identical (Fig. 7). The only difference is observed in the correlation between CC of large-sized herbivores and the timing of the SAI appearance, which is now statistically significant (Fig. 7). Conversely, if the Uluzzian is considered a Neanderthal techno-complex, the correlation between CC of medium-sized herbivores and the end of the NAI is only statistically significant when the FC 1 is used (Fig. 7). However, independently of the biological attribution of the Uluzzian and the incorporation or exclusion of the Châtelperronian, the correlations between CC of small- and medium-sized herbivores and the temporal overlap between NAI and SAI are statistically significant (Fig. 7).

## DISCUSSION

The replacement of Neanderthals by *H. sapiens* was a complex process that differed substantially between regions, which led to the use of the “mosaic” term to describe this biological and cultural transition (*6*, *7*, *30*, *31*). However, framing this mosaic poses substantial challenges, including the scarcity of the archaeological record, uncertainty in some associations between techno-complexes and species, and precision issues in establishing accurate chronologies. These factors have hindered establishing a mechanism for such temporal and spatial patterns. By quantifying the NPP and the herbivore CC across Europe, this study provides evidence supporting the influence of the ecosystems’ CC on the spatiotemporal differences in the timing of the Neanderthal disappearance and their temporal overlap with *H. sapiens*.

Sensitivity tests suggest that the main results are robust concerning the existing uncertainties in the archaeological, chronological, and paleoecological inferences. However, some caveats are necessary. First, the modeling approach to estimate herbivore abundance in this study is based on the bottom-up processes of the food chain regulation, the herbivore guild composition, and the body size of each species, but it does not incorporate other factors that affect herbivore densities (e.g., seasonal resource fluctuations). Moreover, the relationship between NPP and total herbivore biomass might have differed in the past. Consequently, herbivore CC should not be interpreted as a direct estimation of herbivore abundance but rather as an indication of the potential biomass available for secondary consumers in each region (for details, see the “Herbivore CC” section). Recent studies suggest that *H. sapiens* arrived in Western Europe at the beginning of the MIS3 (*8*, *32*, *33*). These archaeological levels were not included in the main analyses because the chronology of these human remains lies beyond the effective range of radiocarbon dating, and the available thermoluminescence dates have large margin errors (for details, see the “Chronological models” section in Materials and Methods). Therefore, the arrival of *H. sapiens* populations in this study should not be interpreted as their first arrival in each region but as spatiotemporal spread patterns between 50 and 30 ka BP. These archaeological sites (Banyoles and Grotte Mandrin) with the earliest evidence of *H. sapiens* in Europe are located in regions (R_14 and R_10) with high and relatively constant ecosystem productivity, where our results indicate an extended period of overlap between both human species.

It has been proposed that Neanderthal populations were smaller and more isolated from one another than *H. sapiens* populations (*34*, *35*), which would imply a higher risk of demographic bottlenecks due to fluctuations in trophic resources. In the context of this study, a longer temporal overlap between the NAI and SAI might indicate a higher probability of interaction between Neanderthals and *H. sapiens*. Still, it should not be interpreted as a continuous and stable coexistence. In this connection, KDEs show that, although NAI or SAI discontinuously occupied most regions during MIS3, both human species likely coexisted in the overlapping areas identified by the BAMs. Besides, in most archaeological sites included in this study, there are discontinuities between the stratigraphic levels with Neanderthal and *H. sapiens* occupations. These discontinuities, along with the alternating presence of Neanderthal and *H. sapiens* at Grotte Mandrin, suggest that spatial segregation might have reduced trophic pressure and facilitated coexistence between both human species at larger geographic scales. It should be noted that local spatial segregation is a common mechanism promoting coexistence among species with similar trophic niches by mitigating direct competition. Consequently, the observed temporal overlap at a regional scale does not necessarily imply coexistence at the local level. Nonetheless, it is worth noting that spatial segregation and low population densities do not negate the competitive exclusion hypothesis. Even if *H. sapiens* and Neanderthals occupied different localities within the same regions, the overlap in this study indicates that they shared the same ecosystems and relied on their productivity.

According to the ecosystem productivity, the biogeographic regions of Europe could be clustered into three main groups: (i) regions with high and constant CC, (ii) regions with high but sharply fluctuating CC, and (iii) regions with low plant or herbivore biomass (Table 1). While the former witnessed a temporal overlap between both human species and thus represented areas of high habitat suitability for humans, the second and third groups correspond to regions prone to experience discontinuities in populations of both human species. Thus, in these regions, Neanderthals disappeared before, or just after, the arrival of *H. sapiens*, while the appearance of SAI took a comparatively more extended time. Accordingly, the ecosystem productivity and food supply may have triggered the spatiotemporal patterns of the MUPT in Europe.

It has long been considered that human population dynamics during MIS3 experienced a “mosaic of depopulation and crowding” (*31*) affected by climatic oscillations. However, the factors that triggered these patterns and the specific regions that could maintain stable human populations throughout MIS3 have not been identified at the European scale. Opposing what is commonly thought, these regions with higher and more constant trophic resources are not exclusively located in the southern European peninsulas (*36*). Thus, some mid-latitude areas, such as the Danube or Rhône basins, featured a high and relatively constant NPP and herbivore CC throughout MIS3, which likely favored the rapid spread of *H. sapiens* and allowed their coexistence with Neanderthals. In contrast, in the meridional sites of the Balkan Peninsula (R_3), we observe the lowest mean values of herbivore CC. Likewise, some regions of the Iberian (R_12 and R_13) and Italian (R_7) peninsulas experienced sharp fluctuations in ecosystem productivity, and therefore, ecological and demographic imbalances were more likely. These results suggest that the regions less affected by the severe climatic fluctuations during MIS3 are not located exclusively in the southern European peninsulas or all the Mediterranean regions.

Analyses of mitochondrial DNA sequences suggest that Neanderthal populations experienced different turnovers before the arrival of *H. sapiens* (*34*, *37*, *38*). On the basis of compared nucleotide diversity estimates, Neanderthal remains from central (R_13) and northern (R_12) Iberia dated to the MIS3 revealed more than sixfold lower diversity than Neanderthals recovered in Eastern Europe (*38*). Likewise, Neanderthal genetic bottlenecks or demographic vacuums have also been proposed for Northern Europe (R_16 and R_5) (*14*), the Carpathian region (R_2) (*15*), and Central and Northern Iberia (*13*, *19*). In all these regions where Neanderthals experienced a loss of genetic diversity or where archaeological data suggest a demographic hiatus before the arrival of *H. sapiens*, the ecosystems’ productivity was low or unstable during MIS3. Moreover, the regions where Neanderthals persisted longer also experienced an earlier arrival of *H. sapiens*. These results suggest that ecosystem CC and its stability throughout MIS3 could be a disruptive agent in the connectivity of Neanderthal populations rather than the appearance of *H. sapiens*. Therefore, these results cast doubt on the contention that the arrival of *H. sapiens* caused the demise of *H. neanderthalensis* in those regions where the NAI disappeared before or shortly after the arrival of the SAI; nonetheless, the competitive exclusion hypothesis (*25*) cannot be ruled out in all regions.

Analyses of *H. sapiens* remains from Peștera cu Oase (R_4) suggest a Neanderthal ancestor four to six generations back in his family tree (*9*). An extended period of contact between *H. sapiens* and Neanderthals in the lower Danube Basin (R_4) is also supported by the chronology of the *H. sapiens* remains recovered from Bacho Kiro (*11*). In France (R_10 and R_11), recent studies suggest that there was a temporal overlap between both species (*8*, *10*). Likewise, in the Mediterranean region of Iberia (R_15), recent discoveries suggest that Neanderthals inhabited this area at a moment when *H. sapiens* had already arrived in the region (*39*, *40*). Results obtained in this study show that in all these regions where Neanderthal genetic continuity and interbreeding with *H. sapiens* have been reported or where recent analyses suggest a longer period of contact between both human species, trophic resources were, on average, markedly higher and more stable. However, in those regions where both human species coexisted, there was substantial variation in the trophic resource availability and the temporal overlap. Correlation tests in this study show that the higher the CC of small- and medium-sized herbivores, the longer the temporal overlap between Neanderthals and *H. sapiens*. Therefore, our results offer a broader picture of the timing of the MUPT in Europe and deepen the knowledge of the factors that generated the demographic patterns inferred from the archaeological and genetic data. The regions characterized by abundant trophic resources displayed a longer temporal overlap between NAI and SAI, which would increase the potential for assimilation between both human species, while likely contributing to a direct or indirect interspecific competition.

The replacement of Neanderthals by our species has been attributed to various factors, such as differential fitness advantages between the two species (due to different reproductive rates or different cognitive, social, or cultural traits) (*41*, *42*), disparities in their population sizes (*43*), or neutral drift (*41*, *44*). Moreover, it has been proposed that interspecific trophic pressure in the carnivore guild probably constrained Neanderthals’ population size (*45*). Results obtained in this study do not directly inform about the biological or cultural traits that potentially contributed to the demise of Neanderthals but confirm that trophic resources played a central role in the spatiotemporal replacement patterns of Neanderthals by *H. sapiens* in Europe.

The spread of invasive species in new environments inevitably affects the food web, increasing the replacement risks of indigenous species (*46*). *H. sapiens* and Neanderthals exploited a wide range of resources, including small-, medium-, and large-sized herbivores (*26*–*28*, *47*–*50*); marine resources (*51*–*53*); and plant foods (*54*, *55*). On the basis of dental wear analyses on fossil specimens, it has been proposed that the proportion of each of these resources in their diet depended on the habitat they inhabited (*56*), and archaeozoological studies suggest that medium-sized herbivores made up the bulk of their diets (*20*, *26*–*28*, *50*). Therefore, their ecological and trophic niches largely overlapped in regions where both species inhabited, making room for interspecific competition. The observed diet variability of both species reveals complex and diversified subsistence strategies, but some authors claim a wider diet breadth for *H. sapiens* compared to Neanderthals (*57*–*59*). If *H. sapiens* included a higher proportion of small-sized herbivores and plant foods, it could explain why the CC of medium-sized herbivores is the only factor influencing the timing of the Neanderthal disappearance, whereas the spread of *H. sapiens* is also significantly affected by other components of the ecosystem’s productivity, such as NPP or the CC of small-sized herbivores. Nevertheless, further archaeozoological and taphonomic analyses at a European scale are still needed to compare the prey spectra, resource diversity, and the proportion of each resource in the diets of *H. sapiens* and Neanderthals.

It is worth noting that correlations do not indicate causality. The results obtained here do not indicate that herbivore CC caused the demise of Neanderthal populations nor their temporal overlap with *H. sapiens*. Conversely, the correlation between CC of small- and medium-sized herbivores and the temporal overlap between Neanderthals and *H. sapiens* suggests that herbivore CC could release the trophic pressure of secondary consumers in some regions, allowing the presence of another species (i.e., *H. sapiens*) in the guild. This idea is supported by the fact that large herbivores (>300 kg) have fewer natural predators (*60*, *61*), and in this study, CC of large-sized ungulates is the only variable systematically uncorrelated with the temporal overlap between both human species. In contrast, top-down effects on small- and medium-sized herbivores (<300 kg) are markedly higher in terrestrial ecosystems (*60*–*62*), so the abundance of small- and medium-sized herbivores affects the richness and stability of the whole secondary consumer guild (*63*, *64*). Hence, herbivore CC during MIS3 likely released the trophic pressure between secondary consumers in some European regions and, therefore, could have slowed down the competitive exclusion processes, affecting the coexistence likelihood between Neanderthals and *H. sapiens*.

The paucity and patchiness of the archaeological record have hampered a general understanding of the processes involved in the replacement patterns of Neanderthals by *H. sapiens*. Besides, it has remained challenging to quantify the effect of the MIS3 climate conditions on the ecosystem productivity and their potential impact on the Neanderthal replacement by modern humans. Data gathered over the past decades and new analytical tools allowed us to incorporate these uncertainties into the analyses and offer robust insights into the mechanisms that affected the MUPT in Europe. Results obtained in this study support the hypothesis that regional differences in the timing of Neanderthal disappearance, the spread patterns of *H. sapiens*, and the temporal coexistence between both human species were affected by trophic resource availability. Thus, this study shows that the ecosystem productivity and stability throughout MIS3 are fundamental for our understanding of the extinction patterns of Neanderthals, the phylogeographic structure of their last populations, and the regional differences in their period of coexistence with our species.

## MATERIALS AND METHODS

### Archaeo-paleontological dataset

A dataset of 1544 dates covering the MIS3 was obtained from the literature. These dates were obtained from 583 archaeo-paleontological levels bearing Mousterian (*n* = 257), Châtelperronian (*n* = 26), Micoquian (*n* = 5), Neronian (*n* = 2), Aurignacian (*n* = 242), Initial Upper Paleolithic (*n* = 27), and Uluzzian (*n* = 9) industries; the remaining levels (*n* = 15) were paleontological. In this study, the term archaeo-paleontological level refers to a specific stratigraphic context from which archaeological or paleontological remains were recovered. The Neronian (*8*), Initial Upper Paleolithic (*11*), Aurignacian (*29*), and Uluzzian (*65*) assemblages have been associated with diagnostic human fossils of *H. sapiens*. Therefore, these techno-complexes were initially grouped under the “*H. sapiens*– associated industries” term (SAI) in this study despite the fact that the entire community does not accept these associations, as discussed below. On the other hand, the Micoquian (*66*), Mousterian, and Châtelperronian (*67*) techno-complexes have been associated with *H. neanderthalensis*, so these techno-complexes were grouped under “Neanderthal-associated industries” (NAI) term. Therefore, in this study, NAI and SAI are only meant to differentiate the techno-complexes made by *H. neanderthalensis* and *H. sapiens*, respectively. The authorship of the Châtelperronian and Uluzzian has been largely debated. Neanderthal remains associated with Châtelperronian lithic tools were recovered from Saint-Césaire and Grotte du Renne (*67*–*69*). However, the Châtelperronian-Neanderthal association has been questioned (*70*). Likewise, two deciduous teeth recovered from Grotta del Cavallo suggest modern human authorship for the Uluzzian (*65*, *71*), but some authors call the reliability of this association into question (*72*). Because the only human remains to yet been recovered from archaeological levels bearing Châtelperronian are those of *H. neanderthalensis* and the only human remains associated with Uluzzian tools were identified as *H. sapiens*, these techno-complexes were included in the main analyses as NAI and SAI, respectively. Still, for the sake of robustness, we performed a sensitivity test by excluding the Châtelperronian from the NAI and the Uluzzian from the SAI categories. Furthermore, the main analyses were also rerun by including the Uluzzian in the NAI category. The techno-complexes that, independently of their morphological affinities, were not recovered from assemblages with diagnostic human fossils [e.g., Szeletian, Bohunician, or Lincombian-Ranisian-Jerzmanowician (LRJ)] were excluded from the NAI or SAI groups.

The dataset also included the herbivore species recovered from 351 archaeo-paleontological levels and their body masses. We included all herbivore species with body weights larger than 1 kg, excluding those below this threshold because of their limited inclusion in traditional archaeozoological analyses, the scarcity of micromammal studies in some regions, and the low trophic pressure exerted by humans on these species. The mean body mass of both sexes for each species was obtained from the PHYLACINE database (*73*). Herbivore carrying capacity was estimated for each herbivore species (for details, see the “Herbivore CC” section). To facilitate data analyses, the herbivore species are commonly grouped into weight size categories. Common weight size classifications used in archaeozoological studies are as follows: small (<20 kg), medium (20 to 100 kg), medium-large (100 to 300 kg), and large-sized (>300 kg) (*20*, *74*). We used this classification but grouped the medium and medium-large sizes into the same category because of two main reasons: first, the representation of medium (20 to 100 kg) and medium-large herbivores (100 to 300 kg) is mainly affected by the specific local settings of each site (e.g., orography); as this study focuses on large biogeographical regions, grouping medium and medium-large herbivores (20 to 300 kg) would avoid these representation skews. Second, Neanderthals and *H. sapiens* had a broad prey spectrum, but medium-sized herbivores with body masses between 20 and 300 kg made the bulk of their diets (*26*, *57*, *75*). Thus, each herbivore species was classified according to the following weight-sized categories: small (<20 kg), medium (20 to 300 kg), and large-sized (>300 kg). The body mass of each species and the specific herbivore guild composition in each region were used in the macroecological modeling approach described below (in the “Herbivore CC” section).

### Chronological models

Uranium-thorium (U/Th), optically stimulated luminescence (OSL), thermoluminescence (TL), and radiocarbon determinations available in the literature were incorporated in the dataset. Regarding the radiocarbon determinations, only measurements obtained with ultrafiltration, Accelerator Mass Spectrometry (AMS), or Acid-Base-Oxidation-Stepped Combustion (ABOx-SC) protocols were retained for analyses. Thus, conventional carbon-14 (^14^C) measurements or problematic chronological determinations with respect to potential contamination, low collagen yield, or dates obtained from burnt bones were included in the dataset but excluded from analyses. All radiocarbon dates were calibrated with the IntCal20 calibration curve. OSL/TL and U/Th chronometric determinations were also incorporated by using the 1Sigma error. Following previous authors, dates with a coefficient of variation equal to or larger than 0.05 were excluded (*76*), and those obtained from marine shells were calibrated with the Marine20 calibration curve (*77*) with a Δ*R* of 0 (*6*). It should be noted that a Δ*R* of 0 was used in previous studies focused on the MIS3 because the marine reservoir effects are unknown for these chronologies and, in terms of overall uncertainty, are less significant at this age range proportionally (*6*, *78*).

Most Paleolithic archaeological assemblages are palimpsests produced by the alternating activity of humans and carnivores. To account for uncertainties in the agent who accumulated the deposits and, therefore, in the chronology of each archaeo-paleontological level, we ran all analyses twice according to two sets of filtering criteria to select the chronometric determinations. First, we used 960 chronometric dates (filtering criteria 1) obtained from different archaeological remains, with or without anthropic modifications, whenever the layers had no stratigraphic issues or discrepancies in their cultural attributions and the chronometric dates met the quality requirements outlined above. The second dataset (filtering criteria 2) consisted of 626 chronometric determinations obtained from archaeological material exclusively brought or modified by humans (e.g., bones with anthropic marks, shell remains, charcoals, etc.). The filtering criteria of the first date-tier are standard in Paleolithic research; however, an increasing number of researchers point out the importance of dating human-modified remains in Paleolithic contexts (*13*, *79*, *80*), so the second date-tier is expected to provide more robust results, albeit the smaller sample size.

In the current study, we built hierarchical BAM with the ChronoModel (CM) software, which has been proposed to be better suited than OxCAL or BCal to reconstruct the timing of archaeological cultures from a regional perspective (*81*). In OxCAL, the Naylor-Smith-Buck-Christen (NSBC) prior places two hyperparameters at the beginning and end of each phase, which pulls the time intervals around the date in a phase with the smallest standard error; on the contrary, CM does not use the NSBC prior and only places hyperparameters at the beginning and end of the entire age model [for details, see (*82*)]. Therefore, using CM yields wider credibility intervals than OxCAL and BCal (*82*). In this study, each “phase” in CM comprises a group of events (i.e., chronological determinations) of a given culture in a particular region. Most Paleolithic assemblages are occupation palimpsests, so chronological measurements obtained from the same archaeological level usually reflect different occupation events. Therefore, each event represents one chronological date associated with a given culture, and multiple chronometric measurements obtained from the same sample (e.g., bone remains) were incorporated into the same event. Following previous studies, we used the default Markov chain Monte Carlo settings, consisting of three chains, 1000 burn iterations, and 500 batch iterations with a maximum of 20 batches and 100,000 acquisition iterations with a thinning interval of 10 (*81*, *82*).

It is important to acknowledge that obtaining precise extinction dates for fossil species or prehistoric cultures is challenging because of the paucity of the archaeological record and various biases and uncertainties that can affect the accuracy of the estimated dates and the archaeological associations of chronometric determinations. While the margin errors of the chronometric dates were directly incorporated into the analyses with permutation tests (for details, see the “Statistical analysis” section), estimating extinction times for fossil species will always be contingent on the availability of new sites, dates, and the integrity of the stratigraphic sequences. Therefore, we performed a further sensitivity test to assess whether the Bayesian age modeling approach could affect the results obtained. Thus, we also used OLE, a widely used methodology to estimate extinction dates from the temporal distribution of species sightings (for details, see Supplementary Methods). Previous studies recommended using each techno-complex’s 5 to 10 oldest and youngest dates to compute the first and last appearance, respectively (*83*, *84*). Therefore, the OLE model was not run in regions with fewer dates than five. We used the median and the range at 95.4% CI of each date obtained with the IntCal20 calibration curve as input. Dates performed on the same archaeological remain were previously combined with the “R_Combine” function in OxCAL. To address the uncertainty of the chronometric determinations, each date from within each of the associated date ranges was randomly drawn from a normal distribution, and this was used instead of the calibrated median dates (*83*). Such a randomly generated set of ages was assessed with the OLE method, and the whole procedure was repeated 10,000 times. We compared the intercepts and the slopes of each correlation between the ecosystem productivity variables and the chronological variables when the latter were obtained from the BAM and OLE models.

Previous studies have used KDE to assess prehistoric demographic changes through the frequency of radiocarbon dates or archaeological sites. Despite the uncertainty of this method as a proxy of human population size (*85*), it is a valuable tool to identify spatiotemporal patterns in human settlements, as their outputs are similar to heatmaps of archaeological site frequencies (*86*). However, whereas simple point density analyses assume that the weight of the point occurs in a single location, KDE spreads the values over an area using a Gaussian distribution (*87*). Therefore, KDE produces smoother visualizations of archaeological site frequencies by mapping distribution between core areas (kernels) and surrounding neighborhoods (*88*).

Once all the procedures described above (filtering criteria of chronometric determination, calibration, BAM, OLE, etc.) were performed, we fitted KDE to assess archaeological site distributions in space and time. We used a bandwidth of 150 km based on the annual migration distance of extant hunter-gatherers (mean, 158.43 ± 2.95) (*89*). The bandwidth should not be interpreted as a yearly mobility radius but as a specific spatial framework that establishes an outer limit to the density estimation (*87*). Accordingly, we generated one raster file with the KDE for the NAI and the SAI from between 50 and 30 ka BP on 1 ka time step. To visually inspect the spatiotemporal overlap patterns between the NAI and the SAI, we multiplied the values of each pixel in the NAI raster by those of the SAI one, so when both were >0, we obtained a value different from 0 that identifies the specific overlap areas (movie S1).

### Paleoclimate reconstruction and validation

The current study used paleoclimate conditions to model vegetation and herbivore population dynamics during MIS3. These climate data were obtained from the Hadley Centre Coupled Model 3 Bristol (HadCM3B) model with active atmosphere, ocean, and sea-ice components (*90*). The HadCM3B general circulation model covers the past 60 ka in the Northern Hemisphere at 0.5° spatial resolution on a monthly time step. This paleoclimate model incorporates the orbital parameters of eccentricity, obliquity and precession, and the global atmospheric CO_2_ concentrations as forcing parameters. Previous studies appraised the accuracy of the HadCM3B simulations with different paleoclimate proxies and other models within the CMIP5 climate model family (*91*). However, assessing the accuracy of these paleoclimate simulations in the specific areas of interest is paramount before using these data for ecological reconstructions.

We performed three tests to assess the accuracy of the HadC3B-M2.1’s simulations in Europe. First, we compared the mean annual temperature (MAT) and precipitation (MAP) estimated in each archaeological site from the HadC3B-M2.1 model against two recent HadCM3 climate simulations (for details, see Supplementary Methods). Second, we assessed whether the simulated climate conditions obtained from the HadC3B-M2.1 model capture the temporal paleoclimate trends observed in long-term records of δO^18^ obtained from seven speleothems in the areas of interest (fig. S2). Last, we estimated temperature and precipitations from 137 palynological assemblages dated to the MIS3 with weighted average regressions and compared these paleoclimate estimations with those obtained from the HadC3B-M2.1 model once a delta bias correction procedure was performed (Supplementary Methods).

Results obtained show that the paleoclimate model used in this study matches more recent models of the HadCM3 climate model family (Supplementary Methods and fig. S3). Moreover, the comparison of the HadC3B-M2.1’s outputs with the δO^18^ values obtained from seven different speleothems suggests that the simulated temperatures and precipitations capture the temporal paleoclimate trends observed in the long-term empirical records recovered from the regions under study (fig. S4). Bias-corrected values of MAT and MAP are in good agreement with the empirical reconstructions made from the palynological record (Supplementary Methods and fig. S5).

### Net primary productivity

NPP was estimated with the Lund-Potsdam-Jena General Ecosystem Simulator (LPJ-GUESS) v.4.0. model. LPJ-GUESS is a process-based dynamic vegetation model, written in C++, that simulates vegetation dynamics in response to climatic and environmental conditions. This model simulates the demographic dynamics of different plant species and, therefore, the structure and composition of the land cover in terms of plant functional types (PFTs) (*92*). PFTs are a set of plant species with similar growth strategies, climatic preferences, leaf phenology, photosynthetic pathways, and life history traits. Age cohorts of PFTs compete for light, water, space, and soil resources in several replicate patches for each simulated grid cell. Different PFTs can be incorporated into the model by specifying their ecological traits and requirements (*93*). In the current study, we used the standard global PFT set described by Smith *et al.* (*94*). The model was run without the nitrogen cycle nor nitrogen limitation because nitrogen deposition is unknown in the Pleistocene. For each grid cell, 75 replicate patches with an area of 0.1 ha were simulated.

In the present study, LPJ-GUESS was run in the “cohort” mode to estimate NPP in the surrounding area of each archaeological and paleontological site that provides information about the herbivore guild composition and the human subsistence strategies during the MUPT. In LPJ-GUESS, NPP is estimated at the end of each simulated year, but the model operates on a daily time step. Monthly paleoclimate drivers can be provided as input, and the model interpolates daily values from the monthly data. Thus, the input climate variables for LPJ-GUESS were monthly temperature (C°), precipitation (mm/month), incoming shortwave radiation (W m^−2^), and rainy days (days/month). These input climate data were obtained from the bias-corrected paleoclimate simulations obtained from the HadCM3B-M2.1 coupled general circulation model (*90*), and therefore, the spatial resolution was inherited from the climate inputs. The model also needs, as input data, the atmospheric carbon dioxide concentration (parts per million) and the specific soil classes for each grid cell to incorporate texture-related variables affecting the hydrology and thermal diffusivity of the soils. The CO_2_ values were obtained from the work of Lüthi *et al.* (*95*), and soil types were obtained from the work of Zobler (*96*). All simulations were initialized with “bare ground” conditions (no biomass), and the model was spun up for 1000 years until the simulated vegetation was in approximate equilibrium. This spin-up phase used monthly temperature, precipitation, incoming shortwave radiation, and rainy days between 55.5 and 54.5 ka BP (*65*). After that, the model was run at a monthly resolution spanning the period comprised between 55 and 30 ka BP.

### Biogeographic regions

Europe hosts different ecosystems, climates, and sets of flora and fauna species. This study specifically examined a geographic region bounded by the latitudes of the ice sheet–free areas during MIS3, spanning from 34°S to 55°N, and longitudinally encompassing the Iberian Peninsula to the Eastern European Plains, ranging from 11°E to 30°W. Regions lying at latitudes covered by ice sheets according to the HadCM3B-M2.1 coupled general circulation model (*77*) cannot be used to estimate NPP; moreover, the number of sites in the Eastern European Plains is extremely low for the MIS3 period, which has been interpreted as the result of the different sort of biases (*97*). To avoid such biases, we selected a geographic extent between 34°S to 55°N and 11°E to 30°W.

The modeling approach used in this study to estimate the biomass of primary consumers depends on the reconstructed NPP and the herbivore guild structure in each biogeographic region. Therefore, we first delimited the biogeographic regions of Europe according to the reconstructed NPP and its evolution throughout MIS3 with a dCORT dissimilarity index (for details, see Supplementary Methods). Two regions can exhibit similar mean NPP values, but their temporal trajectories can vary significantly. To account for this, we used the dCORT index, which compares the raw NPP values and considers the temporal correlation of the NPP series. By incorporating both aspects, the dCORT index comprehensively assess the NPP dynamics across different regions. According to the temporal evolution of each time series of NPP and the proximity between values, there are six main clusters in Europe (Fig. 1). However, some of these clusters cover extensive areas with differences in both the abiotic (e.g., orography, natural barriers, or climate) and various biotic factors, including herbivore guild composition. Consequently, this delimitation of biogeographic regions from NPP was completed with two additional clustering tests.

We divided each cluster obtained from the dCORT index when a difference was observed in the climate conditions or the herbivore guild composition. We used the Köppen-Geiger climate classification, which is based on ranges of temperature and precipitation, to cluster the climate conditions in the surrounding of each archaeological site according to the paleoclimate simulations obtained from the HadCM3B-M2.1 coupled general circulation model (fig. S6) (*90*). We used this paleoclimate model because it shows good correspondence with the empirical paleoclimate reconstructions in the regions of interest (for details, see the “Paleoclimate reconstruction and validation” section). Likewise, we selected the Köppen-Geiger climate classification because, in this study, the accuracy of temperature and precipitation estimations for MIS3 was validated with pollen assemblages, and this classification mainly replies to those climate variables. Climate and NPP groups were considered an initial criterion to differentiate biogeographic regions; nonetheless, two regions with similar climate conditions and similar NPP may harbor different species pools. Therefore, we also assessed the herbivore guild similarity of each region with the Jaccard dissimilarity index (for details, see Supplementary Methods).

It should be noted that the combination of the herbivore guild composition, the paleoclimate conditions, and the temporal trends of NPP as clustering criteria implicitly incorporates the latitudinal, altitudinal, and oceanic gradients that differentiated the ecological settings during MIS3. We identified 16 biogeographic regions across Europe. To avoid denominations based on present-day political/administrative boundaries, they were named consecutively from R_1 to R_16, following an east-west order (for details, see Supplementary Methods).

### Herbivore CC

The herbivore CC in each biogeographic region during MIS3 was estimated according to the modeling approach proposed by (*19*). This approach considers CC as the theoretical maximum population size that a given environment can sustainably support over a specific period and assumes that herbivore abundances depend on the bottom-up processes of the food chain regulation driven by NPP, the allometric relationships between body mass and population density, and the structure and composition of regional herbivore guild communities. In Supplementary Methods, we detail this method as described in (*19*). Therefore, unlike other previous approaches (*98*, *99*), the herbivore abundance in this study depends on the body mass of each herbivore species, the plant biomass, and the herbivore guild composition of each region. However, it should be noted that, despite being rooted in ecological principles of bottom-up processes of the food chain, the relationship between NPP and total herbivore biomass could have differed in the past. Hence, the herbivore abundance in our study should not be understood as a precise estimation of herbivore biomass but rather as the potential biomass of each herbivore species for comparison purposes.

To validate this modeling approach, we obtained data of 674 extant herbivore population densities from a wide range of terrestrial ecosystems worldwide (fig S7). In 92.9% of the national parks/reserves, the estimated herbivore population densities are significantly correlated (*P* < 0.05) with the observed herbivore abundance, with correlation coefficients (*r*) ranging between 0.29 and 0.98. Moreover, when the observed and predicted values were analyzed across all ecosystems, there was a significant positive correlation between the observed and predicted values (*P* < 0.001, *r* = 0.64; fig S8). These results confirm the validity of this macroecological modeling approach.

This modeling approach was applied to each MIS3 herbivore paleocommunity (PCOM) in the biogeographic regions of Europe. A PCOM is determined by the local fauna assemblages (LFAs) found in a specific biogeographic region during a particular period. However, different gaps may affect the number and type of herbivore species that conform to a given PCOM (e.g., uneven sampling effort or taphonomical biases). To assess the totality of the number of species in each PCOM, a rarefaction analysis was performed. Rarefaction (interpolation) and prediction (extrapolation) curves are commonly used to assess species richness according to the sampling effort. This method assumes that species richness (S) approaches an asymptote when the number of samples increases. Accordingly, the observed species richness in each PCOM was compared with the expected richness with a sample size of 100 LFAs in each PCOM, and a bootstrap method was applied (*n* = 500) to obtain the 95% CI for each diversity estimate. Outcomes obtained show that the actual species richness in each PCOM is close to the asymptote (fig. S9), and consequently, increasing the sample size of LFAs would not significantly increase the number of species in each PCOM.

### Statistical analysis

The main purpose of this study is to assess whether the components of the ecosystem CC affected the regional patterns of the MUPT in Europe. Thus, we evaluated whether the end of the NAI, the spread patterns of the SAI, and the overlap between the NAI and SAI techno-complexes in each biogeographic region are correlated with the NPP and the herbivore CC of each region. Accordingly, in this study, we use biogeographic regions as analytical units.

Spatial autocorrelation exists in most geospatial processes because neighboring regions may share some ecological features due to their spatial proximity, which would violate the assumption of independency between observations and could bias the correlation outcomes. Eigenvector spatial filtering (ESF) is a spatial modeling approach widely used in ecology that considers the potential influence of spatial nonindependence between regions. ESF uses a subset of eigenvectors of a spatial weight matrix based on the Moran coefficient and adds them to the original regression model as new independent variables, so the linear combination of these eigenvectors filters the spatial autocorrelation out of the observations, thus enabling model processes to proceed as if the observations were independent (for details, see Supplementary Methods) (*100*).

The dependent (chronological estimations) and independent (NPP and herbivore CC) variables have some degree of uncertainty, which is reflected in their CIs. Previous studies propose using permutation or bootstrap tests for estimating correlation coefficient ranges that incorporate the observed uncertainty in both dependent and independent variables. Hence, the uncertainties related to the chronology, the NPP, and the CC estimations in each region were accounted for by computing the correlation tests with 10,000 random samples drawn from the 95% CI of each dependent and independent variable. Therefore, we randomly permuted the values according to their 95% CIs and fitted a regression model to each permuted set of values, giving us the distribution values that we could expect.

## Supplementary Materials

### This PDF file includes:

Supplementary Text Figs. S1 to S9 Tables S1 and S2 Legend for movie S1 References
