# Supplementary material for: Neanderthal coexistence with Homo sapiens in Europe was affected by herbivore carrying capacity
Source: Sci Adv. 2023 Sep 22;9(38):eadi4099. doi: 10.1126/sciadv.adi4099 (PMC10516502; doi:10.1126/sciadv.adi4099)
Supplement: Supplementary file 1 — Supplementary Text Figs. S1 to S9 Tables S1 and S2 Legend for movie S1 References [file sciadv.adi4099_sm.pdf]

Science Advances
AAAS

Supplementary Materials for

Neanderthal coexistence with *Homo sapiens* in Europe was affected by herbivore carrying capacity

Marco Vidal-Cordasco *et al.*

Corresponding author: Ana B. Marín-Arroyo, marinab@unican.es; Marco Vidal-Cordasco, vidalma@unican.es

Sci. Adv. **9**, eadi4099 (2023)
DOI: 10.1126/sciadv.adi4099

The PDF file includes:

Supplementary Text
Figs. S1 to S9
Tables S1 and S2
Legend for movie S1
References

Other Supplementary Material for this manuscript includes the following:

Movie S1

Supplementary Text

Biogeographic regions

We first delimited the biogeographic regions of Europe according to the reconstructed NPP and its evolution throughout MIS3 with a dCORT dissimilarity index (*101*). The dCORT index is widely used to compare both the raw values and the temporal correlation of two series of data (S_1 and S_2):
(1)

$$CORT(S_1, S_2) = \frac{\sum_{i=1}^{p-1}\left(u_{(i+1)} - u_i\right)\left(v_{(i+1)} - v_i\right)}{\sqrt{\sum_{i=1}^{p-1}\left(u_{(i+1)} - u_i\right)^2}\sqrt{\sum_{i=1}^{p-1}\left(v_{(i+1)} - v\right)^2}}$$

Where u and v are the raw values of the series S_1 and S_2, respectively, and p is the length of each series. CORT (S_1, S_2) belongs to the interval (-1,1). A CORT (S_1, S_2) value of 1 indicates a positive correlation, whereas a CORT value of -1 indicates a negative correlation. In contrast, a CORT (S_1, S_2) value of 0 indicates that the temporal trends of S_1 and S_2 are independent. To consider not only the temporal correlation between each pair of time series but also the proximity between values, we computed the dCORT (S_1, S_2) index as follows:
(2)

$$dCORT(S_1, S_2) = f[CORT(S_1, S_2)] * d(S_1, S_2)$$

Where f(x) is:
(3)

$$f(x) = \frac{2}{1 + \exp(k\,x)}, k \geq 0$$

Following previous studies (*19*), the value of k was 2 (*101*). This function was used to compare the reconstructed NPP of each archaeological and paleontological site during MIS3 across Europe. Fig. 1 shows the results obtained.

According to the temporal evolution of each time series of NPP and the proximity between values, there are six main clusters in Europe. In northern Iberia and southwestern France, we observe one cluster characterised by high NPP that experienced sharp fluctuations from ~44 ka years BP onwards (dark blue colour in Fig. 1). Much more stable was the NPP in the mid-latitude Mediterranean regions of Europe (light blue colour in Fig. 1) and along some relevant river basins (Danube or Ebro basins). In Central and Northern Europe, NPP experienced a decreasing trend throughout MIS3, with significant drops in plant biomass (magenta colour in Fig. 1). This decreasing trend was more meaningful in the highest altitude areas, where we observe the lowest NPP values, probably affected by the aggradation of permafrost during MIS3 (brown colour in Fig. 1). In the sites located in the southerly areas of the Balkan Peninsula (red colour in Fig. 1) NPP was significantly lower than in the coastal Mediterranean sites of Europe (green colour in Fig. 1), where NPP was relatively constant. Therefore, according to the dCORT index, the NPP classification generates six clusters that broadly correspond with present-day biogeographic regions. Yet, these clusters cover extensive areas with differences abiotic and biotic conditions.

Consequently, this delimitation of biogeographic regions from NPP was completed with two additional clustering tests. First, we divided each cluster obtained from the dCORT index when a difference was observed in the climate conditions according to the Köppen-Geiger Climate Classification. Thus, the ranges of temperatures and precipitations were used to classify the climate conditions surrounding each archaeo-paleontological site according to the paleoclimate simulations obtained from the HadCM3B-M2.1 coupled general circulation model (*90*). Secondly, we assessed

the similarity in the herbivore guild composition between regions with the Jaccard Dissimilarity Index (JDI).

The JDI is based on presence-absence data of the herbivore species, and it is calculated as follows: (4)

$$JDI = 1 - \frac{c}{(a + b + c)}$$

Where *c* is the number of species shared between both areas, and *a* and *b* are the number of species only present in one of the regions. The lower the JDI, the more similar the herbivore composition guild of both regions is. We used the presence/absence data instead of the abundance of each taxon because the Minimum Number of Individuals (MNI) and Number of Identified Specimens (NISP) would be biased not only by taphonomic factors but also by the accumulator agents, the duration of the occupation events, etc. Moreover, MNI and NISP provide valuable information, but these values are not systematically reported in the zooarchaeological studies. To avoid these potential sources of bias, this study focused on the presence/absence of each taxon. Below, we briefly summarise the results obtained from the classification criteria to delimitate these biogeographic regions.

In the Balkan Peninsula, we identified three regions: R_1, along the Lower Danube basin, R_2 around the Carpathians, and R_3 in the southernmost areas. R_1 had a humid continental climate similar to that observed in the present-day hemiboreal regions, with mean annual temperatures colder than nowadays (Supplementary Fig. 6), which is in close agreement with recent empirical temperature estimations in archaeological sites from this area (*102*). Precipitations in the R_1 were lower and temperatures higher than in R_2, where the climate was affected by the latitude and the orography (Supplementary Fig. 6). Thus, R_2 had a wetter and colder climate, which is supported by recent paleoenvironmental studies (*103*). On the contrary, in R_3 the climate conditions were significantly warmer and drier (Supplementary Fig. 6) due to the lower latitudes and the proximity of the Mediterranean Sea. According to the JDI, the herbivore guild composition differed significantly between the R_1, R_2 and R_3 regions (Fig. 2): while R_1 hosted a diverse herbivore guild of all sizes, the number of large-sized herbivores was significantly lower in R_3 than in R_1 and R_2 (Fig. 2).

Unlike the Lower Danube basin (R_1), the Upper Danube had lower temperatures and precipitations. Moreover, despite their geographic proximity, the herbivore guild composition significantly differed between R_1 and R_4. Likewise, according to the Köppen-Geiger Climate Classification, Central and Northern Europe (R_5) had a subarctic climate, with long cold winters and short cool summers. These climatic estimations for MIS3 agree with recent paleoclimatic reconstructions (*104*). The herbivore guild composition also differed between R_4 and R_5: in R_5 we observe more species adapted to colder (e.g. *Obives moschatus*, *Saiga tatarica*) and drier (e.g. *Equus hydruntinus*) conditions than in R_4 (Fig. 2).

In the Italian Peninsula, Croatia and Serbia, the coldest and wettest conditions are observed in the Prealpine region of R_7 (Supplementary Fig. 6). In the south of R_7, the proximity to the sea and the lower altitudes contributed to the warmer conditions of the Adriatic (R_6) and Ligurian (R_9) regions. According to the Köppen–Geiger Classification, the R_6 and R_9 had similar climate conditions; nonetheless, the herbivore guild composition differed significantly between both regions (Fig. 2). Likewise, the Tyrrhenian (R_8) had a different herbivore guild composition and featured drier summers and milder winters due to the lower latitudes. In France, mean annual precipitations were lower in R_11 than in R_10, despite both regions having similar annual temperatures. On the other hand, the herbivore guild composition also differed between R_11 and R_10: the former host a higher number of large-sized herbivores. However, the main difference

between R_11 and R_10 is the temporal trends of NPP (Fig. 1). In the Iberian Peninsula, the R_12 is characterised by annual temperatures ~5 °C lower than nowadays, which is in agreement with recent paleoclimate reconstructions (*105*, *106*). Two different climatic belts can be observed in the Mediterranean area: the Thermomediterranean region (R_15), affected by the proximity to the coast and the lower latitudes, and the Mesomediterranean (R_14). Between R_12 and R_14 there is a transitional area (R_13) with lower temperatures and precipitations. The herbivore guild composition did not differ between R_12 and R_13, but NPP was significantly lower, probably due to the higher altitude of this inner area. According to the Jaccard Dissimilarity Index, the herbivore guild composition significantly differed between the remaining regions of the Iberian Peninsula (Fig. 2).

<u>Optimal Linear Estimations (OLE)</u>

Optimal Linear Estimations (OLE), also known as Weibull extreme value model, estimate the extinction/origin date from a temporal distribution of species sightings (for details, see (*19*, *84*)). OLE uses a number of temporally ordered dates ($T_1 > T_2 > \ldots > T_k$), where k is the most recent record when estimating the origin timing and the first known date to estimate extinction time (*107*). The OLE model assumes that the origin/extinction time ($\hat{\theta}$) has the form of a weighted sum of the date times:

(5)

$$\hat{\theta} = \sum_{i=1}^{k} a_i T_{n-i+1}$$

Where the weight vector above (a) is given by:

(6)

$$a = (e^t \wedge^{-1} e)^{-1} \wedge^{-1} e$$

Where e is a vector of k 1's and $\wedge$ the symmetric k x k matrix with element:

(7)

$$\wedge_{ij} = \frac{(\Gamma(2\hat{v} + i)\Gamma(\hat{v} + j))}{(\Gamma(\hat{v} + i)\Gamma(j))} \; j \leq i$$

where Γ is the standard gamma function and v^ is an estimate of the shape parameter of the Weibull extreme value distribution given by:

(8)

$$\hat{v} = \frac{1}{k-1} \sum_{i-1}^{k-2} log \frac{T_n - T_{n-k+1}}{T_n - T_{i+1}}$$

T_n being the n times a species is observed over the period of time T (*108*). Thus, if a species is assumed to be extinct, the upper bound of $1 - \alpha$ confidence interval for θ is:

(9)

$$S_U = \frac{T_n - c(\alpha) T_{n-k+1}}{1 - c(\alpha)},$$

Where

(10)

$$c(\alpha) = \left(\frac{k}{-log\ \alpha}\right)^{-\hat{v}}.$$

These functions to estimate the first and last appearance of each culture in the biogeographic regions under study were applied with the R software package sExtinct (*108*). Following the

recommendations of previous studies (*83*, *84*), we used the 5-10 oldest dates of each technocomplex to compute the first appearance of each culture in a specific region. Thus, OLE models were not run in regions where the number of dates was lower than 5. We used the median and the range at 95.4% CI of each date obtained with the IntCal20 calibration curve as input, and dates performed on the same archaeological remain were previously combined with the "R_Combine" function in OxCAL. To assess the uncertainty of the chronometric determinations, each date from within each of the associated date ranges was randomly drawn from a normal distribution and this was used instead of the calibrated median dates (*83*). Such randomly generated set of ages was assessed with the OLE method, and the whole procedure was repeated 10,000 times. The R codes data used to perform these analyses are available from Zenodo (https://doi.org/10.5281/zenodo.8215395)

Paleoclimate validation

The accuracy of the HadC3B-M2.1's simulations in Europe was assessed with three tests. We first compared the mean annual temperature (MAT) and precipitation (MAP) estimated in each archaeological site from the HadC3B-M2.1 model against two recent HadCM3 climate simulations (*109*, *110*). Then, we compared the climate conditions obtained from the HadC3B-M2.1 model with the temporal paleoclimate trends observed in long-term records of δO^{18} obtained from seven speleothems in the areas of interest. It should be noted that δO^{18} in speleothems is related to annual temperatures and rainfalls, so this proxy is commonly used to assess whether paleoclimate simulations capture key qualitative dynamics observed in the empirical data (*111*). However, it is still necessary to quantitatively evaluate the accuracy of the paleoclimate predictions. To this end, we estimated temperature and precipitations from palynological assemblages dated to the MIS3 and compared these paleoclimate estimations with those obtained from the HadC3B-M2.1 model once a bias correction procedure was performed. Recent studies have demonstrated that, among various paleoclimate bias correction procedures, the delta method yields better results for the HadAM3H atmospheric model (*112*). The delta method corrects simulated paleoclimatic data by applying the difference between present-day observed and simulated climate to past simulated climate (for details see (*112*). Thus, the estimated MAT and MAP values from the palynological record were compared with the MAT and MAP obtained from the HadCM3B-M2.1 coupled general circulation model after using the delta correction method. Present-day values of MAT and MAP have been obtained from the Climate Research Unit v.4 (CRU) dataset (*113*).

Following previous studies, we used weighted averaging (WA) regressions to perform pollen-based paleoclimate reconstructions. The WA method assumes a unimodal response of plant species along environmental gradients to estimate paleoclimate by weighting taxon abundances from target assemblages using the calibrated optima (*114*). Thus, predictive functions to calculate MAT and MAP were derived from a training set of modern pollen taxa obtained from the Eurasian Modern Pollen Database (EMPD) v.270 (*115*). This training set was used to obtain temperature and precipitation transfer functions based on pollen subsets using WA regression techniques. Before computing the predictive functions, the assemblages with low pollen counts (< 100 grains) were discarded and excluded all non-terrestrial pollen taxa. This filtering step is commonly used because terrestrial and aquatic taxa are unevenly affected by climate (*116*). Lastly, taxonomy for analysis was standardised for all taxa to a common resolution at the genus level. A higher taxonomic resolution cannot always be achieved in the palynological record. Prediction errors were simulated by bootstrap cross-validation (n of boot cycles: 500). Then, the predictive functions were applied to the fossil pollen record (SI Appendix, Table S2).

We obtained the percentage of each pollen taxa recovered from 137 palynological assemblages dated to the MIS3 from the literature. Each percentage was obtained from the species count or the published palynological diagrams with the DigitPlot software. Before estimating MAT and MAP, some filtering criteria were also applied to this dataset. First, only palynological assemblages with chronologies between 55 and 30 ka cal BP were used in the analysis. Pollen assemblages with less than 100 pollens and species with low representation (<5%) were excluded. Non-terrestrial pollen taxa, including ferns and non-pollen palynomorphs, were not retained for analysis. Lastly, the percentages of each taxon were re-adjusted and the predictive functions were applied to estimate the MAT and MAP in each sample. The correlation coefficient to estimate MAT was 0.76 and the root-mean-square error (RMSE) was 4.26. For MAP estimation, the correlation was 0.6 and the RMSE 273.1 (SI Appendix, Table S2).

A significant positive correlation (p-value ≤0.05) can be observed between the climate outputs obtained from (*90*) and those obtained from (*109*) and (*110*), with determination coefficients (r) ranging between 0.72 and 0.89 (SI Appendix, Fig S3). These results show that the paleoclimate model used in this study matches more recent models of the HadCM3 climate model family. The comparison of the HadC3B-M2.1's outputs with the δO^{18} values obtained from seven different speleothems suggests that the simulated temperatures and precipitations capture the temporal paleoclimate trends observed in the long-term empirical records recovered from the regions under study (SI Appendix, Fig S4). Therefore, these analyses suggest that the HadCM3B circulation model reflects the temporal and spatial trends in the paleoclimate values during MIS3 in the regions of interest (SI Appendix, Fig S4). Lastly, the mean difference between the observed and predicted values of MAT during MIS3 in the archaeological sites with pollen samples was -0.05 °C on average with a root-mean-square error (RMSE) of 2.95. The difference between the observed and predicted values of MAP was 3.06 mm/month on average, with an RMSE of 115.06. Besides, there is a positive correlation between the observed and predicted values of MAT (p-value <0.01, r^2 0.91) and MAP (p-value >0.01, r^2 0.79) (SI Appendix, Fig S5). Therefore, bias-corrected values of MAT and MAP are in good agreement with the empirical reconstructions made from the palynological record.

Herbivore carrying capacity

The biomass of a herbivore population (B) is commonly estimated by multiplying its population density (D) by the mean adult body mass of the species. Accordingly, the total biomass (THB) of all herbivore species in a given ecosystem could be expressed as follows:
(11)

$$THB = \sum_{i=1}^{n} D_i * W_i$$

Where n is the number of herbivore species in the community, D_i is the population density of species i expressed in ind/km^2, and W is the mean body mass of both sexes in kg. Damuth (*117*) demonstrated that population density changes allometrically with body size across different ecosystems:
(12)

$$D_i = c\, W_i^{-3/4}$$

Where D is expressed in ind/km^2, c is a constant and W is the mean body mass in kg. Thus, D can be substituted in equation (12):

(13)

$$THB = \sum_{i=1}^{n} (c\, W_i^{-3/4}) * W_i$$

Furthermore, *c* can be estimated as follows:
(14)

$$c = \frac{\text{THB}}{\sum_{i=1}^{n} W_i^{1/4}}$$

Therefore, to obtain the *c* value, THB should be first estimated. Previous studies showed that the total herbivore biomass that could be sustained in a given ecosystem depends on NPP (*118–121*). Drawing on these previous studies, (*19*) provided a predictive equation obtained from data gathered from a wide range of terrestrial ecosystems to estimate THB from NPP:
(15)

$$\log_{10} THB = 1.401 * \log_{10} NPP - 0.642$$

Where both THB and NPP are expressed in g/m^2/yr. Accordingly, the biomass (B) of a specific herbivore population species (*i*) in a given ecosystem can be estimated with equation (16):
(16)

$$B_i = D_i * W_i \rightarrow \left(\frac{\text{THB}}{\sum_{i=1}^{n} W_i^{1/4}} W_i^{-3/4} \right) * W_i$$

This modelling approach has been previously validated (*19*). In the current study, we extended that validation with a larger sample size of herbivore densities (*61*, *122*). Thus, we obtained data of 674 extant herbivore population densities from a wide range of terrestrial ecosystems (SI Appendix, Fig S7). In 92.9% of the national parks/reserves, the estimated herbivore population densities are significantly correlated (p-value <0.05) with the observed herbivore abundance, with correlation coefficients (r) ranging between 0.29 and 0.98. Moreover, when the observed and predicted values were analysed across all ecosystems, there was a significant positive correlation between the observed and predicted values (p-value <0.001, r. 0.64) (SI Appendix, Fig S8).

To assess the completeness of the faunal assemblages in each biogeographic region, a rarefaction analysis was performed. Rarefaction curves assess the species richness according to the sampling effort. Thus, we compared the observed species richness in each region with the expected richness with a sample size of 100 faunal assemblages, and the bootstrap method was applied (n=500) to obtain the 95% CI for each diversity estimate. In SI Appendix, Fig S9 shows the outcomes obtained, which suggest that increasing the sample size of fauna assemblages would not significantly increase the number of species in each region. The R codes and data used to perform these analyses are available from All data needed to evaluate the conclusions in the paper are present in the article or available from GitHub (https://github.com/ERC-Subsilience/Data-and-code-associated-with-Neanderthal-coexistence-with-Homo-sapiens-in-Europe) and Zenodo (https://doi.org/10.5281/zenodo.8215395)

Eigenvector Spatial Filtering

Spatial filtering methods isolate spatial effects in regression models to account for spatial autocorrelation. The Eigenvector Spatial Filtering (ESF) uses the eigenvector of a transformed

spatial link matrix to overcome the autocorrelation issues in regression models (*100*). The regression can be expressed as follows:
(17)

$$Yi = \sum_{k=1}^{n} X_{i,k}\beta_k + \mathrm{f}\,MC\,(s_i) + \varepsilon_i\,,\ \ \varepsilon_i{\sim}N(0,\sigma^2)\,¡$$

Where Y is the dependent variable observed at i-th sample into trend $\sum_{k=1}^{n} X_{i,k}\beta_k$, spatial process $\mathrm{f}\,MC\,(s_i)$ depending on location s_i and noise ε_i (*123*). The incorporation of the spatial structure into the regression model eliminates the residual spatial dependence and estimates regression coefficients (β_k) appropriately (*123*). In this study, ESF defined $fMC(s_i)$ using the MC-based spatial process to eliminate residual spatial dependence with weighted sum of the Moran eigenvectors. These analyses were performed with the *spmoran* R package (*123*). The R codes and data used to perform these analyses are available from GitHub (https://github.com/ERC-Subsilience/Data-and-code-associated-with-Neanderthal-coexistence-with-Homo-sapiens-in-Europe) and Zenodo (https://doi.org/10.5281/zenodo.8215395)

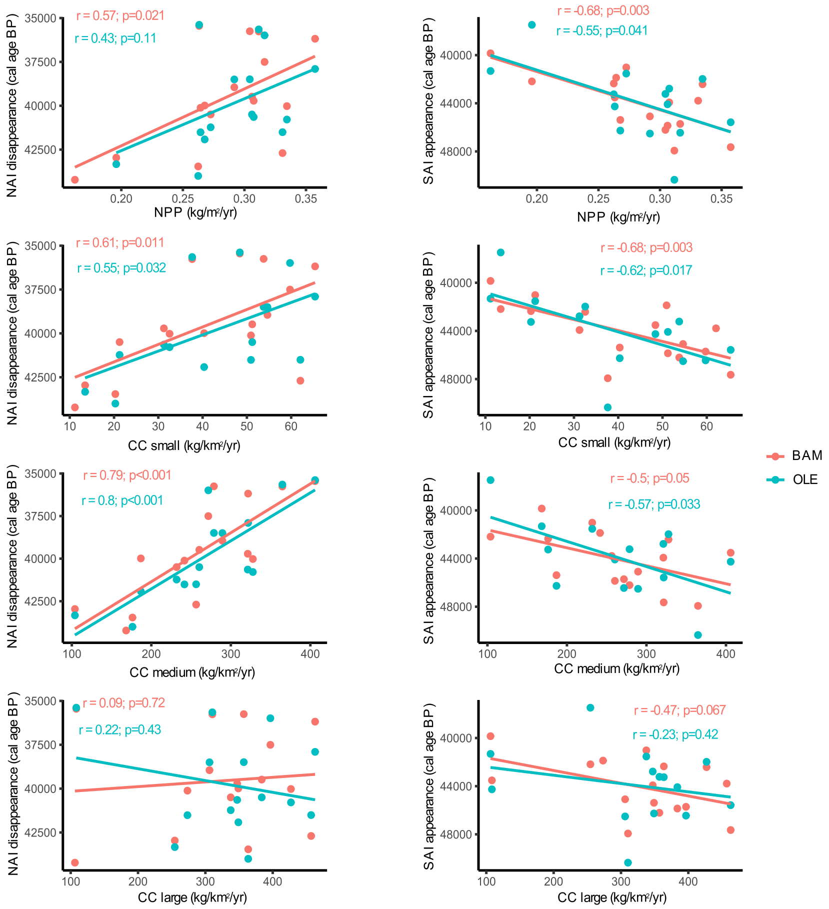

Fig. S1. Correlations with OLE and BAM.

Comparison of the linear correlations between NPP and herbivore carrying capacity (CC) with the timing of the NAI (Neanderthal Affiliated Industries) disappearance and the SAI (*H. sapiens* Affiliated Industries) appearance in each region according to Bayesian age models (BAM) and optimal linear estimations (OLE).

Table S1. Comparison of chronological models.

Comparison of the intercepts and slopes of the models run with chronology obtained from the Bayesian Age Models (BAM) and those obtained with Optimal Linear Estimations (OLE) according to the ANOVA.

Dependent variable	Independent variable		BAM	OLE	p-value	F-ratio
End NAI	NPP	Intercept	48767.6	48480.4	0.557	0.35
		Slope	-32431.9	-29604.2	0.894	0.02
	CC small	Intercept	43618.0	43780.9	0.583	0.31
		Slope	-99.5906	-92.351	0.889	0.02
	CC medium	Intercept	47104.0	47417.7	0.476	0.52
		Slope	-28.7449	-28.2118	0.950	0.00
	CC large	Intercept	40417.8	37519.4	0.802	0.06
		Slope	-2.65872	6.73	0.405	0.71
Start SAI	NPP	Intercept	35012.3	34708.9	0.949	0.00
		Slope	31777.2	32664.5	0.957	0.00
	CC small	Intercept	40342.4	39715.8	0.891	0.02
		Slope	90.6213	109.135	0.691	0.16
	CC medium	Intercept	40125.3	38390.6	0.864	0.03
		Slope	14.9165	20.9255	0.590	0.30
	CC large	Intercept	40578.3	41712.4	0.946	0.00
		Slope	10.5902	6.89461	0.702	0.15
Overlap	NPP	Intercept	-8640.49	-8231.43	0.917	0.01
		Slope	47593.7	45638.5	0.956	0.00
	CC small	Intercept	-1352.4	-2306.2	0.876	0.02
		Slope	152.727	181.178	0.729	0.12
	CC medium	Intercept	-4776.84	-4390.96	0.909	0.01
		Slope	36.7731	34.7744	0.906	0.01
	CC large	Intercept	2541.46	7861.85	0.920	0.01
		Slope	7.17292	-8.21586	0.418	0.68

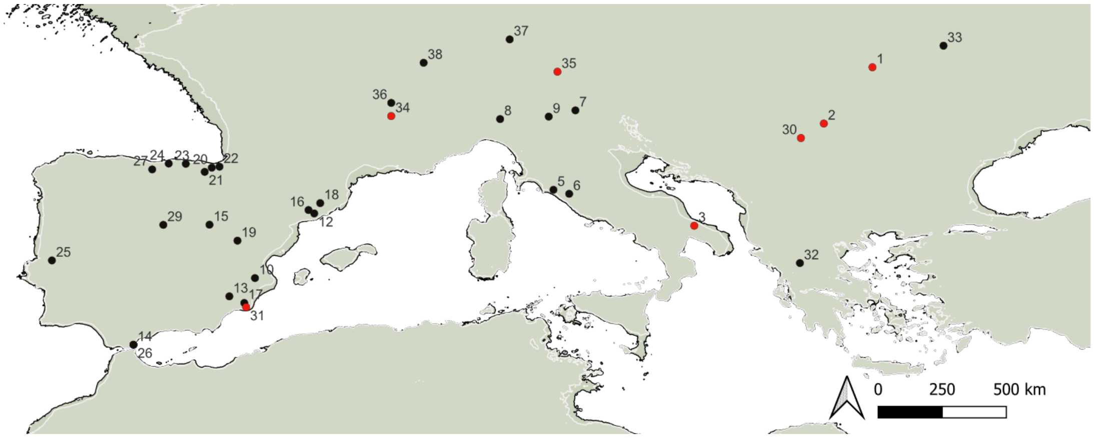

Fig. S2. Paleoclimate proxies for validation.
In black colour, sites with palynological samples used in the WA regressions. In red colour, localities with speleothems dated to the MIS3.

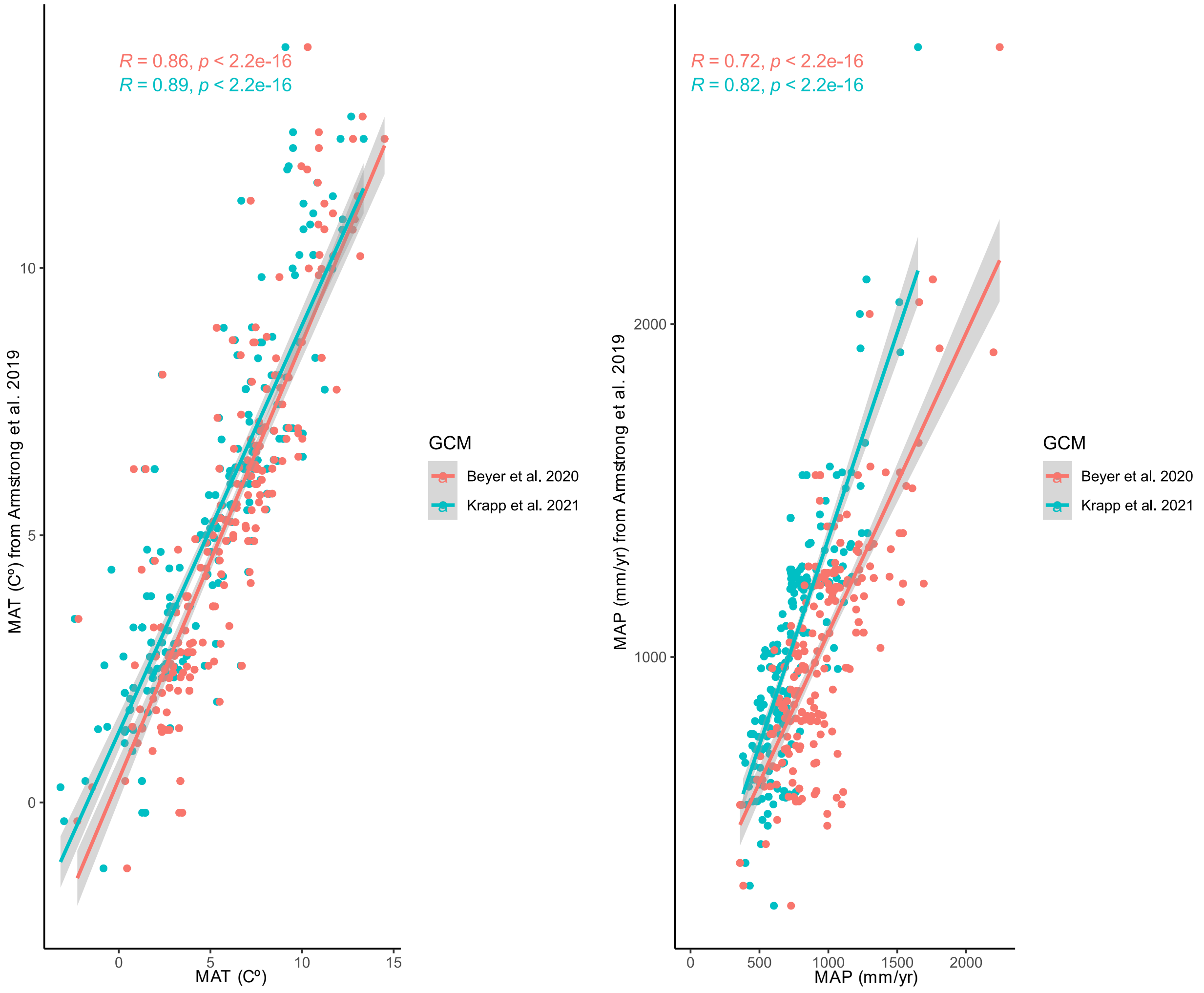

Fig. S3. Comparison of HadCM3B-M2.1 circulation models.
Correlation between Mean Annual Temperature (MAT) and Precipitation (MAP) of the HadCM3B-M2.1 circulation model and two more recent paleoclimate simulations. Dots represent each archaeo-paleontological site included in this study.

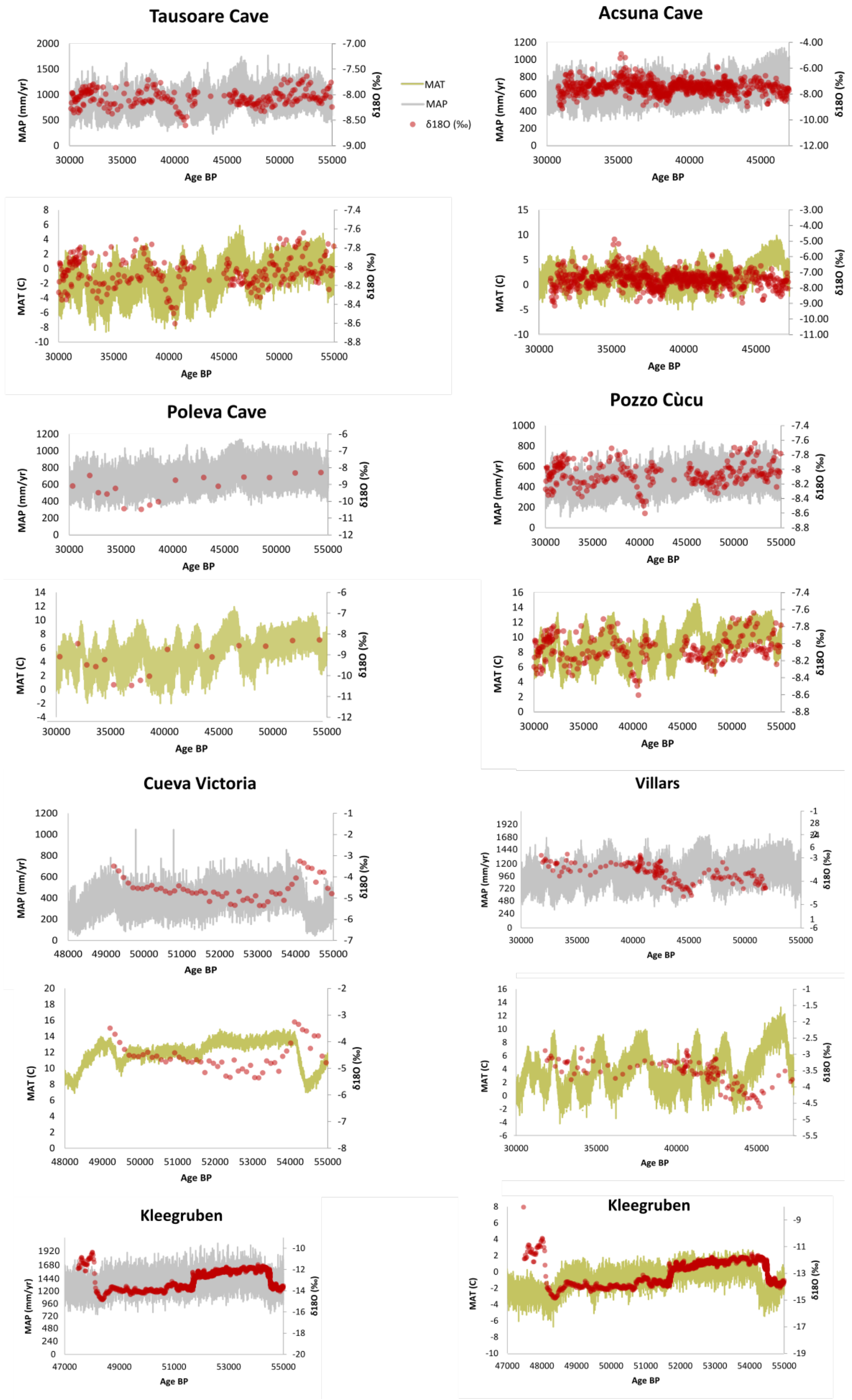

Fig. S4. Paleoclimate validation with δO18 records from speleothems.

Comparison of the simulated precipitation and temperature obtained from the HadC3B-M2.1 against the δO^{18} records obtained from five speleothems. For details, see Fig. S2.

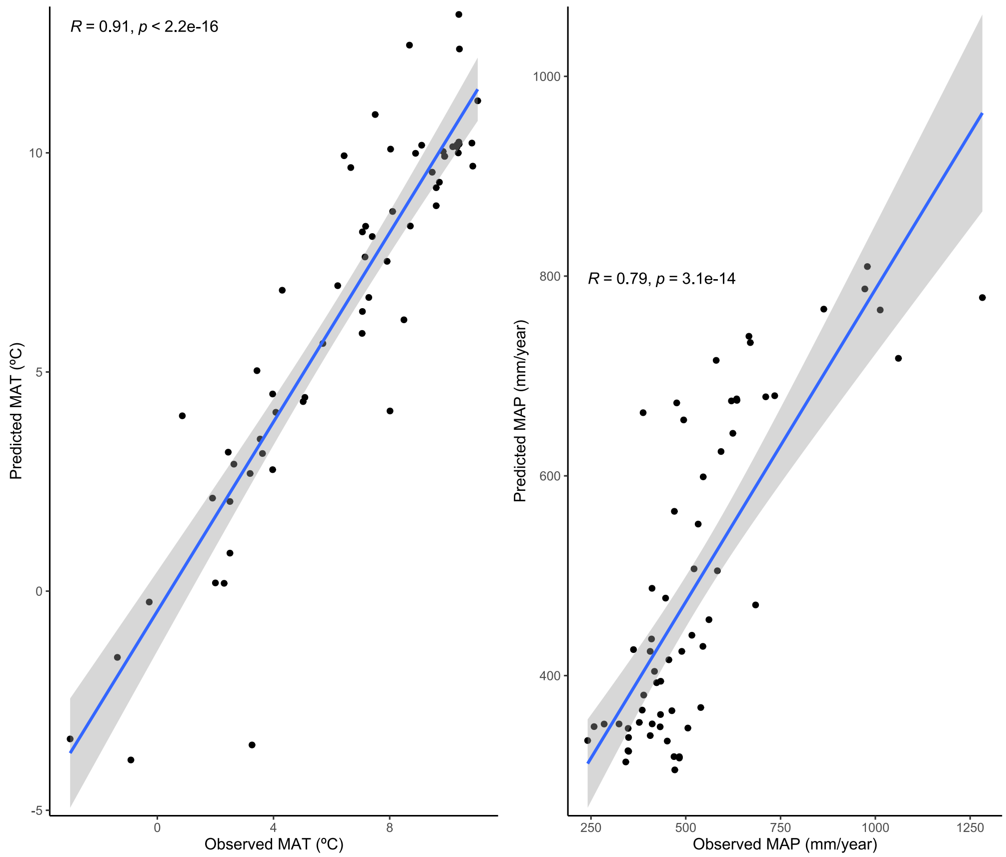

Fig. S5. Paleoclimate validation with the palynological record.
Correlation between the estimated Mean Annual Temperatures (observed MAT) and Precipitation (observed MAP) from the palynological record and the values obtained from the HadCM3B-M2.1 coupled general circulation model in each archaeo-paleontological site.

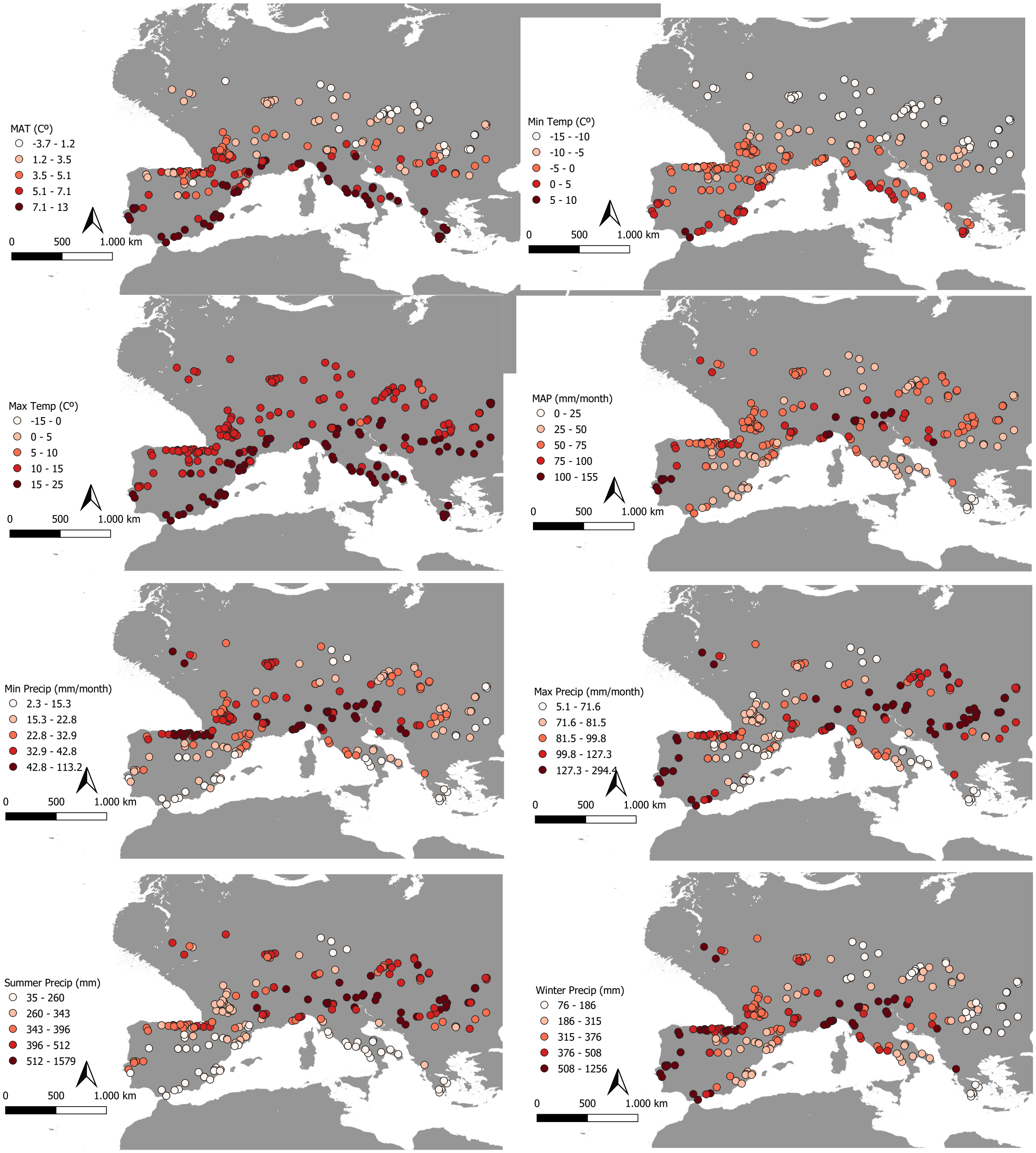

Fig. S6. Paleoclimate conditions for the Köppen–Geiger climate classification.
Range of temperature (C°) and precipitation (mm/month) values during MIS3 in each archaeo-palaeontological site to perform the Köppen–Geiger climate classification. All climate variables were obtained from Armstrong et al. (*90*) after bias correction (see the "Paleoclimate reconstruction and validation" section for details).

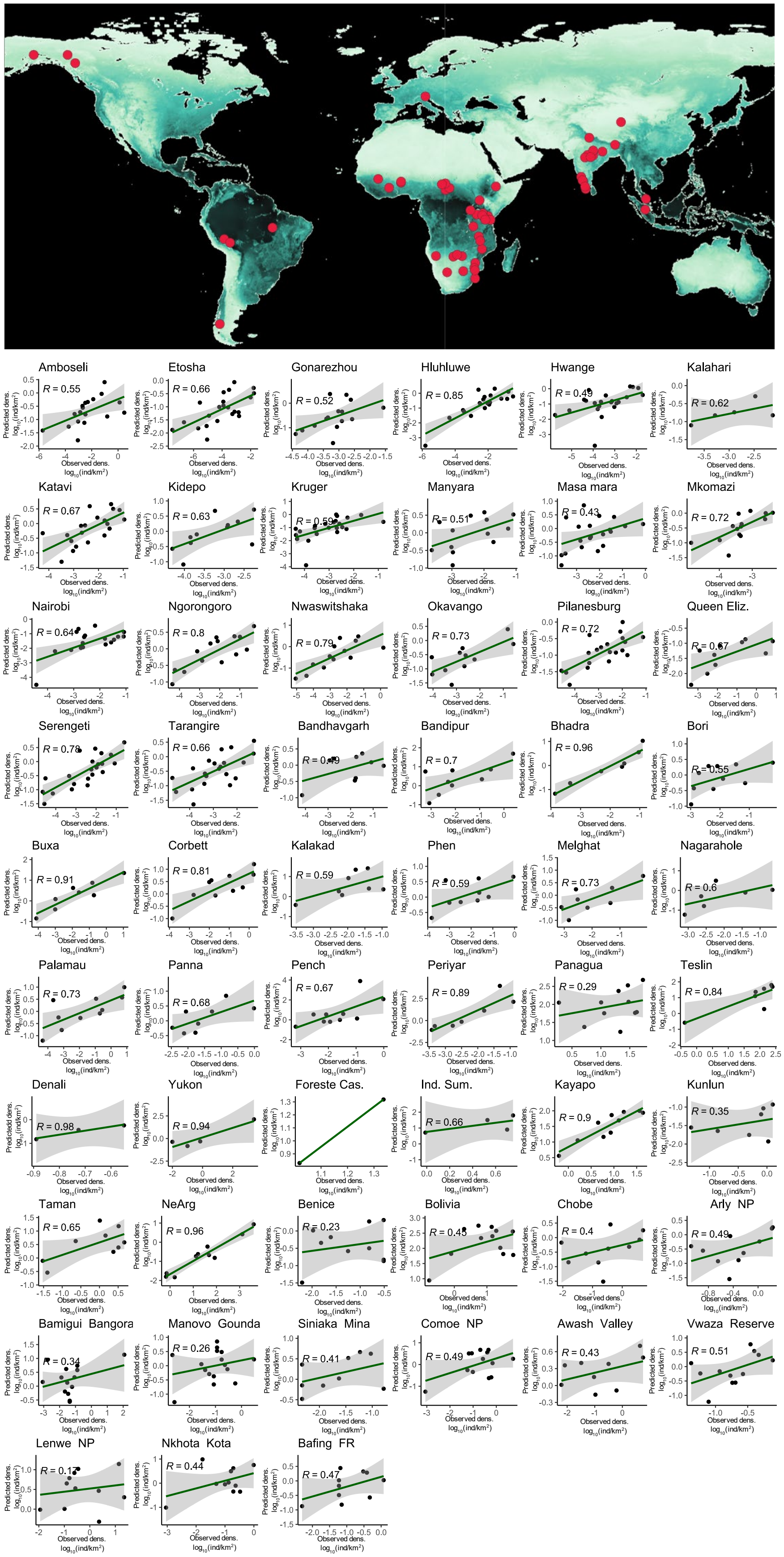

Fig. S7. Validation of herbivore abundance estimates in different ecosystems. Geographical distribution of the national parks or reserves (red dots) with data on extant herbivore population densities used to validate the model. Below is the correlation between the observed and predicted population density values of extant herbivore species in different national parks/reserves. The mean values are represented with black dots, and the 95% CI of the regression model is shaded grey.

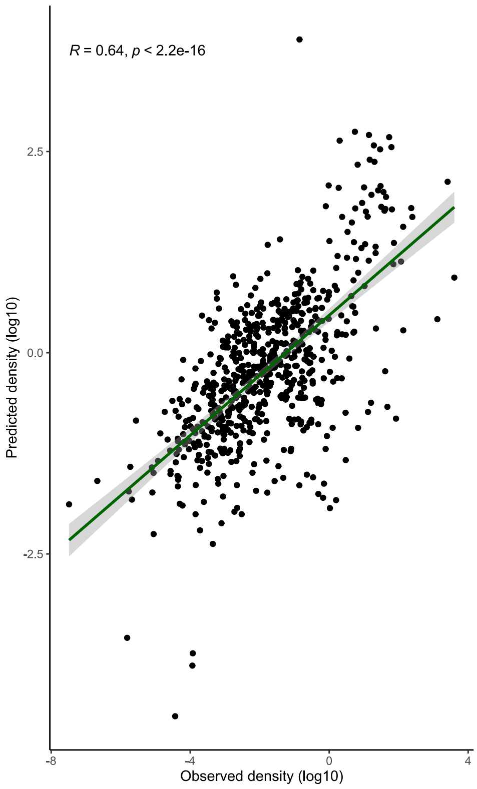

Fig. S8. Validation of herbivore abundance estimates worldwide.
Two-sided Pearson's correlation coefficient test used to assess the relationship between the observed and predicted values of herbivore densities across all the national parks/reserves.

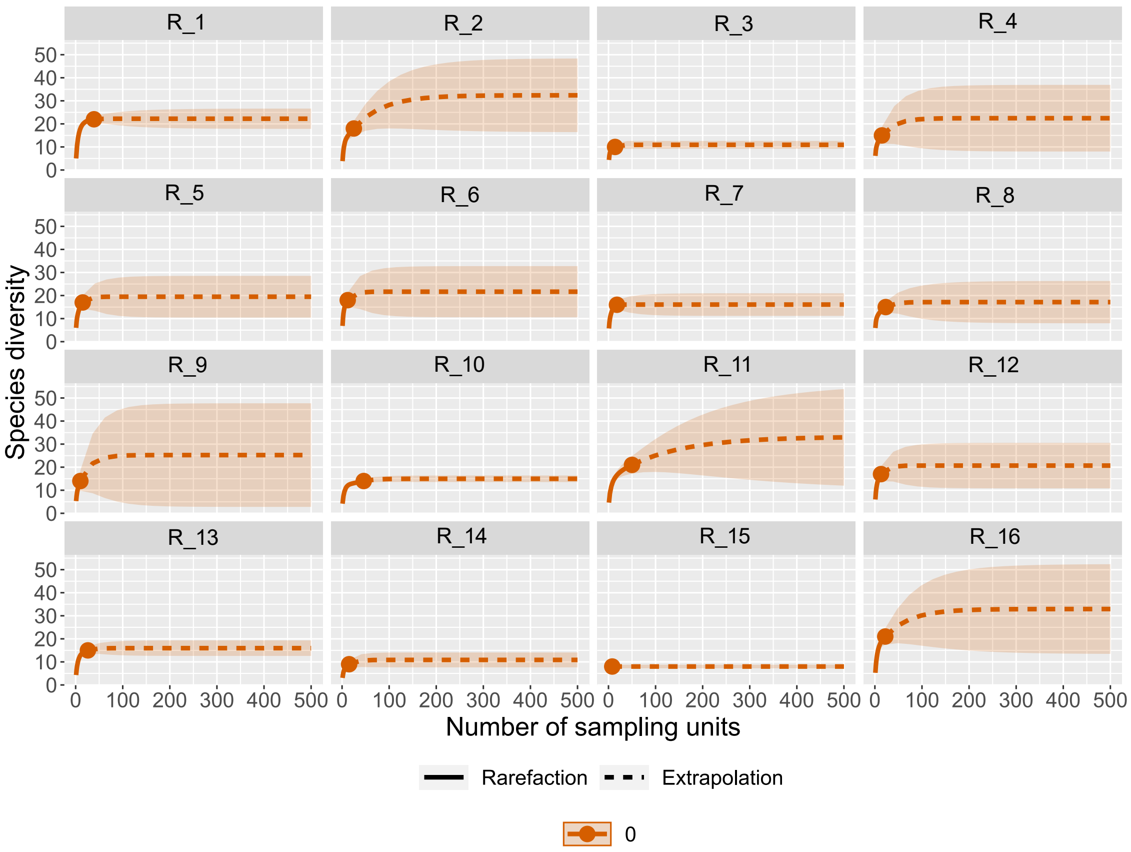

Fig. S9. Rarefaction test.

Species diversity in each biogeographic region (brown dot) according to the LFAs used in the study and expected increase in the species diversity (discontinuous line) if the sample of LFAs was 500

Table S2. Paleoclimate predictive model based on pollen samples.

Values of Root Mean Square Error of Prediction (RSEMP), correlation coefficient (r^2), p-values (p), average bias of the model residuals and maximum bias of the model residuals after the bootstrap cross-validation process.

**Mean Annual Temperature (C°)**					**Mean Annual Precipitation (mm/year)**				
RMSE	r^2	p	Avg. Bias	Max. Bias	RMSE	r^2	p	Avg. Bias	Max. Bias
4.26	0.76	<0.001	-0.05	8.5	273	0.60	0.001	-21	784.2

Movie S1 (separate file). Spatiotemporal replacement patterns of Neanderthals by *H. sapiens.*
Spatiotemporal distribution of archaeological sites associated with NAI (Neanderthal Affiliated Industries) and SAI (*H. sapiens* Affiliated Industries) techno-complexes according to Kernel Density Estimates on a 1k years timestep.

REFERENCES

1. C. Darwin, *On the Origin of Species by Means of Natural Selection Or the Preservation of Favoured Races in the Struggle for Life* (Oxford Univ. Press, 1859).

2. G. Hardin, The competitive exclusion principle. *Science* **131**, 1292–1297 (1960).

3. A. D. Letten, P. J. Ke, T. Fukami, Linking modern coexistence theory and contemporary niche theory. *Ecol. Monogr.* **87**, 161–177 (2017).

4. W. J. Ripple, R. L. Beschta, Large predators limit herbivore densities in northern forest ecosystems. *Eur. J. Wildl. Res.* **58**, 733–742 (2012).

5. A. D. Barnosky, Megafauna biomass tradeoff as a driver of Quaternary and future extinctions. *Proc. Natl. Acad. Sci. U.S.A.* **105**, 11543–11548 (2008).

6. T. Higham, K. Douka, R. Wood, C. B. Ramsey, F. Brock, L. Basell, M. Camps, A. Arrizabalaga, J. Baena, C. Barroso-Ruíz, C. Bergman, C. Boitard, P. Boscato, M. Caparrós, N. J. Conard, C. Draily, A. Froment, B. Galván, P. Gambassini, A. Garcia-Moreno, S. Grimaldi, P. Haesaerts, B. Holt, M. J. Iriarte-Chiapusso, A. Jelinek, J. F. Jordá Pardo, J. M. Maíllo-Fernández, A. Marom, J. Maroto, M. Menéndez, L. Metz, E. Morin, A. Moroni, F. Negrino, E. Panagopoulou, M. Peresani, S. Pirson, M. De La Rasilla, J. Riel-Salvatore, A. Ronchitelli, D. Santamaria, P. Semal, L. Slimak, J. Soler, N. Soler, A. Villaluenga, R. Pinhasi, R. Jacobi, The timing and spatiotemporal patterning of Neanderthal disappearance. *Nature* **512**, 306–309 (2014).

7. L. G. Straus, Neanderthal last stand? Thoughts on Iberian refugia in late MIS 3. *J. Quat. Sci.* **37**, 283–290 (2022).

8. L. Slimak, C. Zanolli, T. Higham, M. Frouin, J.-L. Schwenninger, L. J. Arnold, M. Demuro, K. Douka, N. Mercier, G. Guérin, H. Valladas, P. Yvorra, Y. Giraud, A. Seguin-Orlando, L. Orlando, J. E. Lewis, X. Muth, H. Camus, S. Vandevelde, M. Buckley, C. Mallol, C. Stringer, L. Metz, Modern human incursion into Neanderthal territories 54,000 years ago at Mandrin, France. *Sci. Adv.* **8**, eabj9496 (2022).

9. Q. Fu, M. Hajdinjak, O. T. Moldovan, S. Constantin, S. Mallick, P. Skoglund, N. Patterson, N. Rohland, I. Lazaridis, B. Nickel, B. Viola, K. Prüfer, M. Meyer, J. Kelso, D. Reich, S. Pääbo, An early modern human from Romania with a recent Neanderthal ancestor. *Nature* **524**, 216–219 (2015).

10. I. Djakovic, A. Key, M. Soressi, Optimal linear estimation models predict 1400–2900 years of overlap between Homo sapiens and Neandertals prior to their disappearance from France and northern Spain. *Sci. Rep.* **12**, 15000 (2022).

11. J. J. Hublin, N. Sirakov, V. Aldeias, S. Bailey, E. Bard, V. Delvigne, E. Endarova, Y. Fagault, H. Fewlass, M. Hajdinjak, B. Kromer, I. Krumov, J. Marreiros, N. L. Martisius, L. Paskulin, V. Sinet-Mathiot, M. Meyer, S. Pääbo, V. Popov, Z. Rezek, S. Sirakova, M. M. Skinner, G. M. Smith, R. Spasov, S. Talamo, T. Tuna, L. Wacker, F. Welker, A. Wilcke, N. Zahariev, S. P. McPherron, T. Tsanova, Initial Upper Palaeolithic *Homo sapiens* from Bacho Kiro Cave, Bulgaria. *Nature* **581**, 299–302 (2020).

12. P. R. Nigst, P. Haesaerts, F. Damblon, C. Frank-Fellner, C. Mallol, B. Viola, M. Götzinger, L. Niven, G. Trnka, J. J. Hublin, Early modern human settlement of Europe north of the alps occurred 43,500 years ago in a cold steppe-type environment. *Proc. Natl. Acad. Sci. U.S.A.* **111**, 14394–14399 (2014).

13. A. B. Marín-Arroyo, J. Rios-Garaizar, L. G. Straus, J. R. Jones, M. de la Rasilla, M. R. González Morales, M. Richards, J. Altuna, K. Mariezkurrena, D. Ocio, Chronological reassessment of the Middle to Upper Paleolithic transition and Early Upper Paleolithic cultures in Cantabrian Spain. *PLOS ONE* **13**, e0194708 (2018).

14. W. Roebroeks, J.-J. Hublin, K. MacDonald, Continuities and discontinuities in Neandertal presence: A closer look at Northwestern Europe. *Dev. Quat. Sci.* **14**, 113–123 (2011).

15. M. Staubwasser, V. Drăgușin, B. P. Onac, S. Assonov, V. Ersek, D. L. Hoffmann, D. Veres, Impact of climate change on the transition of Neanderthals to modern humans in Europe. *Proc. Natl. Acad. Sci. U.S.A.* **115**, 9116–9121 (2018).

16. S. O. Rasmussen, M. Bigler, S. P. Blockley, T. Blunier, S. L. Buchardt, H. B. Clausen, I. Cvijanovic, D. Dahl-Jensen, S. J. Johnsen, H. Fischer, V. Gkinis, M. Guillevic, W. Z. Hoek, J. J. Lowe, J. B. Pedro, T. Popp, I. K. Seierstad, J. P. Steffensen, A. M. Svensson, P. Vallelonga, B. M. Vinther, M. J. C. Walker, J. J. Wheatley, M. Winstrup, A stratigraphic framework for abrupt climatic changes during the Last Glacial period based on three synchronized Greenland ice-core records: Refining and extending the INTIMATE event stratigraphy. *Quat. Sci. Rev.* **106**, 14–28 (2014).

17. M. Fernández-García, M. Vidal-Cordasco, J. R. Jones, A. B. Marín-Arroyo, Reassessing paleoenvironment conditions during Middle-to-Upper Paleolithic transition in the Cantabrian region (Southwestern Europe). *Quat. Sci. Rev.* **301**, 107928 (2023).

18. J. Ochando, G. Amorós, J. S. Carrión, S. Fernández, M. Munuera, J. Camuera, G. Jiménez-Moreno, P. González-Sampériz, F. Burjachs, A. B. Marín-Arroyo, M. Roksandic, C. Finlayson, Iberian Neanderthals in forests and savannahs. *J. Quat. Sci.*, **37**, 335–362 (2022).

19. M. Vidal-Cordasco, D. Ocio, T. Hickler, A. B. Marín-Arroyo, Ecosystem productivity affected the spatiotemporal disappearance of Neanderthals in Iberia. *Nat. Ecol. Evol.* **6**, 1644–1657 (2022).

20. A. B. Marín-Arroyo, G. Terlato, M. Vidal-Cordasco, M. Peresani, Subsistence of early anatomically modern humans in Europe as evidenced in the Protoaurignacian occupations of Fumane Cave, Italy. *Sci. Rep.* **13**, 3788 (2023).

21. J. S. Carrión, J. Ochando, S. Fernández, R. Blasco, J. Rosell, M. Munuera, G. Amorós, I. Martín-Lerma, S. Finlayson, F. Giles, R. Jennings, G. Finlayson, F. Giles-Pacheco, J. Rodríguez-Vidal, C. Finlayson, Last Neanderthals in the warmest refugium of Europe: Palynological data from Vanguard Cave. *Rev. Palaeobot. Palynol.* **259**, 63–80 (2018).

22. J. Riel-Salvatore, F. Negrino, G. Pothier Bouchard, A. Vallerand, S. Costa, S. Benazzi, The ‘Semi-Sterile Mousterian’ of Riparo Bombrini: Evidence of a late-lasting Neanderthal refugium in Liguria. *J. Quat. Sci.* **37**, 268–282 (2022).

23. R. W. Dennell, M. Martinón-Torres, J. M. Bermúdez de Castro, Hominin variability, climatic instability and population demography in Middle Pleistocene Europe. *Quat. Sci. Rev.* **30**, 1511–1524 (2011).

24. A. Timmermann, K. S. Yun, P. Raia, J. Ruan, A. Mondanaro, E. Zeller, C. Zollikofer, M. Ponce de León, D. Lemmon, M. Willeit, A. Ganopolski, Climate effects on archaic human habitats and species successions. *Nature* **604**, 495–501 (2022).

25. W. E. Banks, F. d'Errico, A. T. Peterson, M. Kageyama, A. Sima, M. F. Sánchez-Goñi, Neanderthal extinction by competitive exclusion. *PLOS ONE* **3**, e3972 (2008).

26. A. B. Marín-Arroyo, A. Sanz-Royo, What Neanderthals and AMH ate: Reassessment of the subsistence across the Middle–Upper Palaeolithic transition in the Vasco-Cantabrian region of SW Europe. *J. Quat. Sci.*, **37**, 320–334 (2022).

27. W. Rendu, S. Renou, M. C. Soulier, S. Rigaud, M. Roussel, M. Soressi, Subsistence strategy changes during the Middle to Upper Paleolithic transition reveals specific adaptations of human populations to their environment. *Sci. Rep.* **9**, 15817 (2019).

28. M. Romandini, J. Crezzini, E. Bortolini, P. Boscato, F. Boschin, L. Carrera, N. Nannini, A. Tagliacozzo, G. Terlato, S. Arrighi, F. Badino, C. Figus, F. Lugli, G. Marciani, G. Oxilia, A. Moroni, F. Negrino, M. Peresani, J. Riel-Salvatore, A. Ronchitelli, E. E. Spinapolice, S. Benazzi, Macromammal and bird assemblages across the late Middle to Upper Palaeolithic transition in Italy: An extended zooarchaeological review. *Quat. Int.* **551**, 188–223 (2020).

29. J. J. Hublin, The modern human colonization of western Eurasia: When and where? *Quat. Sci. Rev.* **118**, 194–210 (2015).

30. R. Mora, J. Martínez-Moreno, M. Roy Sunyer, A. Benito Calvo, A. Polo-Díaz, S. Samper Carro, Contextual, technological and chronometric data from Cova Gran: Their contribution to discussion of the Middle-to-Upper Paleolithic transition in northeastern Iberia. *Quat. Int.* **474**, 30–43 (2018).

31. W. Davies, D. White, M. Lewis, C. Stringer, Evaluating the transitional mosaic: Frameworks of change from Neanderthals to Homo sapiens in eastern Europe. *Quat. Sci. Rev.* **118**, 211–242 (2015).

32. B. A. Keeling, R. Quam, I. Martínez, J. L. Arsuaga, J. Maroto, Reassessment of the human mandible from Banyoles (Girona, Spain). *J. Hum. Evol.* **174**, 103291 (2023).

33. K. Prüfer, C. Posth, H. Yu, A. Stoessel, M. A. Spyrou, T. Deviese, M. Mattonai, E. Ribechini, T. Higham, P. Velemínský, J. Brůžek, J. Krause, A genome sequence from a modern human skull over 45,000 years old from Zlatý kůň in Czechia. *Nat. Ecol. Evol.* **5**, 820–825 (2021).

34. S. Castellano, G. Parra, F. A. Sánchez-Quinto, F. Racimo, M. Kuhlwilm, M. Kircher, S. Sawyer, Q. Fu, A. Heinze, B. Nickel, J. Dabney, M. Siebauer, L. White, H. A. Burbano, G. Renaud, U. Stenzel, C. Lalueza-Fox, M. De La Rasilla, A. Rosas, P. Rudan, D. Brajkoviæ, Ž. Kucan, I. Gušic, M. V. Shunkov, A. P. Derevianko, B. Viola, M. Meyer, J. Kelso, A. M. Andrés, S. Pääbo, Patterns of coding variation in the complete exomes of three Neandertals. *Proc. Natl. Acad. Sci. U.S.A.* **111**, 6666–6671 (2014).

35. J. C. French, *Palaeolithic Europe: A Demographic and Social Prehistory* (Cambridge Univ. Press, 2021).

36. M. Carvalho, N. Bicho, Complexity in the Middle to Upper Paleolithic transition in Peninsular Southern Europe and application of refugium concepts. *J. Quat. Sci.* **37**, 380–393 (2022).

37. B. Vernot, E. I. Zavala, A. Gómez-Olivencia, Z. Jacobs, V. Slon, F. Mafessoni, F. Romagné, A. Pearson, M. Petr, N. Sala, A. Pablos, A. Aranbur, J. M. B. De Castro, E. Carbonell, B. Li, M. T. Krajcarz, A. I. Krivoshapkin, K. A. Kolobova, M. B. Kozlikin, M. V. Shunkov, A. P. Derevianko, B. Viola, S. Grote, E. Essel, D. L. Herraéz, S. Nagel, B. Nickel, J. Richter, A. Schmidt, B. Peter, J. Kelso, R. G. Roberts, J. L. Arsuaga, M. Meyer, Unearthing Neanderthal population history using nuclear and mitochondrial DNA from cave sediments. *Science* **372**, eabf1667 (2021).

38. L. Dalén, L. Orlando, B. Shapiro, M. Brandström-Durling, R. Quam, M. T. P. Gilbert, J. C. Díez Fernández-Lomana, E. Willerslev, J. L. Arsuaga, A. Götherström, Partial genetic turnover in Neandertals: Continuity in the East and population replacement in the West. *Mol. Biol. Evol.* **29**, 1893–1897 (2012).

39. J. Zilhão, The late persistence of the Middle Palaeolithic and Neandertals in Iberia: A review of the evidence for and against the "Ebro Frontier" model. *Quat. Sci. Rev.* **270**, 107098 (2021).

40. J. I. Morales, A. Cebrià, A. Burguet-Coca, J. L. Fernández-Marchena, G. García-Argudo, A. Rodríguez-Hidalgo, M. Soto, S. Talamo, J. M. Tejero, J. Vallverdú, J. M. Fullola, The Middle-to-Upper Paleolithic transition occupations from Cova Foradada (Calafell, NE Iberia). *PLOS ONE* **14**, e0215832 (2019).

41. D. R. Shultz, M. Montrey, T. R. Shultz, Comparing fitness and drift explanations of Neanderthal replacement. *Proc. R. Soc. B* **286**, 20190907 (2019).

42. A. R. Vahdati, J. D. Weissmann, A. Timmermann, M. Ponce de León, C. P. E. Zollikofer, Exploring Late Pleistocene hominin dispersals, coexistence and extinction with agent-based multi-factor models. *Quat. Sci. Rev.* **279**, 107391 (2022).

43. K. Vaesen, F. Scherjon, L. Hemerik, A. Verpoorte, Inbreeding, Allee effects and stochasticity might be sufficient to account for Neanderthal extinction. *PLOS ONE* **14**, e0225117 (2019).

44. O. Kolodny, M. W. Feldman, A parsimonious neutral model suggests Neanderthal replacement was determined by migration and random species drift. *Nat. Commun.* **8**, 1040 (2017).

45. S. E. Churchill, *Thin on the Ground: Neandertal Biology, Archeology and Ecology* (Wiley Blackwell, 2014).

46. P. David, E. Thébault, O. Anneville, P. F. Duyck, E. Chapuis, N. Loeuille, Impacts of invasive species on food webs. *Adv. Ecol. Res.* **56**, 1–60 (2017).

47. E. Discamps, J. Jaubert, F. Bachellerie, Human choices and environmental constraints: Deciphering the variability of large game procurement from Mousterian to Aurignacian times (MIS 5-3) in southwestern France. *Quat. Sci. Rev.* **30**, 2755–2775 (2011).

48. J. E. Fa, J. R. Stewart, L. Lloveras, J. M. Vargas, Rabbits and hominin survival in Iberia. *J. Hum. Evol.* **64**, 233–241 (2013).

49. M. Carvalho, T. Peireira, C. Manso, Rabbit exploitation in the Middle Paleolithic at Gruta Nova da Columbeira, Portugal. *J. Archaeol. Sci. Rep.* **21**, 821–832 (2018).

50. D. C. Salazar-García, R. C. Power, A. Sanchis Serra, V. Villaverde, M. J. Walker, A. G. Henry, Neanderthal diets in central and southeastern Mediterranean Iberia. *Quat. Int.* **318**, 3–18 (2013).

51. C. B. Stringer, J. C. Finlayson, R. N. E. Barton, Y. Fernández-Jalvo, I. Cáceres, R. C. Sabin, E. J. Rhodes, A. P. Currant, J. Rodríguez-Vidal, F. Giles-Pacheco, J. A. Riquelme-Cantal, Neanderthal exploitation of marine mammals in Gibraltar. *Proc. Natl. Acad. Sci. U.S.A.* **105**, 14319–14324 (2008).

52. M. Cortés-Sánchez, A. Morales-Muñiz, M. D. Simón-Vallejo, M. C. Lozano-Francisco, J. L. Vera-Peláez, C. Finlayson, J. Rodríguez-Vidal, A. Delgado-Huertas, F. J. Jiménez-Espejo, F. Martínez-Ruiz, M. A. Martínez-Aguirre, A. J. Pascual-Granged, M. M. Bergadà-Zapata, J. F. Gibaja-Bao, J. A. Riquelme-Cantal, J. A. López-Sáez, M. Rodrigo-Gámiz, S. Sakai, S. Sugisaki, G. Finlayson, D. A. Fa, N. F. Bicho, Earliest known use of marine resources by Neanderthals. *PLOS ONE* **6**, e24026 (2011).

53. J. Zilhão, D. E. Angelucci, M. Araújo Igreja, L. J. Arnold, E. Badal, P. Callapez, J. L. Cardoso, F. d'Errico, J. Daura, M. Demuro, M. Deschamps, C. Dupont, S. Gabriel, D. L. Hoffmann, P. Legoinha, H. Matias, A. M. Monge Soares, M. Nabais, P. Portela, A. Queffelec, F. Rodrigues, P. Souto, Last Interglacial Iberian Neandertals as fisher-hunter-gatherers. *Science* **367**, eaaz7943 (2020).

54. A. G. Henry, A. S. Brooks, D. R. Piperno, Plant foods and the dietary ecology of Neanderthals and early modern humans. *J. Hum. Evol.* **69**, 44–54 (2014).

55. M. Mariotti Lippi, B. Aranguren, S. Arrighi, D. Attolini, S. Benazzi, F. Boschin, S. Florindi, A. Moroni, F. Negrino, P. Pallecchi, L. Pisaneschi, J. Riel-Salvatore, A. Ronchitelli, A. Revedin, New evidence of plant food processing in Italy before 40ka. *Quat. Sci. Rev.* **312**, 108161 (2023).

56. L. Fiorenza, S. Benazzi, A. Estalrrich, O. Kullmer, Diet and cultural diversity in Neanderthals and modern humans from dental macrowear analyses. *Dent. Wear Evol. Biocultural Context.*, 39–72 (2020).

57. M. P. Richards, E. Trinkaus, Isotopic evidence for the diets of European Neanderthals and early modern humans. *Proc. Natl. Acad. Sci. U.S.A.* **106**, 16034–16039 (2009).

58. M. Ne Patou-Mathis, Neanderthal subsistence behaviours in Europe. *Int. J. Osteoarchaeol.* **10**, 379–395 (2000).

59. S. Münzel, N. J. Conard, Change and continuity in subsistence during the Middle and Upper Palaeolithic in the Ach Valley of Swabia (South-west Germany). *Int. J. Osteoarchaeol.* **14**, 225–243 (2004).

60. A. R. E. Sinclair, S. Mduma, J. S. Brashares, Patterns of predation in a diverse predator–prey system. *Nature* **425**, 288–290 (2003).

61. I. A. Hatton, K. S. McCann, J. M. Fryxell, T. J. Davies, M. Smerlak, A. R. E. Sinclair, M. Loreau, The predator-prey power law: Biomass scaling across terrestrial and aquatic biomes. *Science* **349,** aac6284 aac6284 (2015).

62. G. Rodríguez-Gómez, G. H. Cassini, P. Palmqvist, M. S. Bargo, N. Toledo, J. A. Martín-González, N. A. Muñoz, R. F. Kay, S. F. Vizcaíno, Testing the hypothesis of an impoverished predator guild in the Early Miocene ecosystems of Patagonia: An analysis of meat availability and competition intensity among carnivores. *Palaeogeogr. Palaeoclimatol. Palaeoecol.* **554**, 109805 (2020).

63. P. J. den Boer, The present status of the competitive exclusion principle. *Trends Ecol. Evol.* **1**, 25–28 (1986).

64. J. G. Owen, On Productivity as a predictor of rodent and carnivore diversity. *Ecology* **69**, 1161–1165 (1988).

65. A. Moroni, A. Ronchitelli, S. Arrighi, D. Aureli, S. Bailey, P. Boscato, F. Boschin, G. Capecchi, J. Crezzini, K. Douka, G. Marciani, D. Panetta, F. Ranaldo, S. Ricci, S. Scaramucci, V. Spagnolo, S.

Benazzi, P. Gambassini, Grotta del Cavallo (Apulia-Southern Italy). The Uluzzian in the mirror. *J. Anthropol. Sci.* 125–160 (2018).

66. W. J. Rink, H. P. Schwarcz, K. Valoch, L. Seitl, C. B. Stringer, ESR dating of Micoquian industry and Neanderthal remains at Kůlna Cave, Czech Republic. *J. Archaeol. Sci.* **23**, 899–901 (1996).

67. S. E. Bailey, J. J. Hublin, Dental remains from the Grotte du Renne at Arcy-sur-Cure (Yonne). *J. Hum. Evol.* **50**, 485–508 (2006).

68. J. J. Hublin, F. Spoor, M. Braun, F. Zonneveld, S. Condemi, A late Neanderthal associated with Upper Palaeolithic artefacts. *Nature* **381**, 224–226 (1996).

69. F. Welker, M. Hajdinjak, S. Talamo, K. Jaouen, M. Dannemann, F. David, M. Julien, M. Meyer, J. Kelso, I. Barnes, S. Brace, P. Kamminga, R. Fischer, B. M. Kessler, J. R. Stewart, S. Pääbo, M. J. Collins, J.-J. Hublin, Palaeoproteomic evidence identifies archaic hominins associated with the Châtelperronian at the Grotte du Renne. *Proc. Natl. Acad. Sci. U.S.A.* **113**, 11162–11167 (2016).

70. B. Gravina, F. Bachellerie, S. Caux, E. Discamps, J. P. Faivre, A. Galland, A. Michel, N. Teyssandier, J. G. Bordes, No reliable evidence for a Neanderthal-Châtelperronian association at La Roche-à-Pierrot, Saint-Césaire. *Sci. Rep.* **8**, 15134 (2018).

71. S. Benazzi, K. Douka, C. Fornai, C. C. Bauer, O. Kullmer, J. Svoboda, I. Pap, F. Mallegni, P. Bayle, M. Coquerelle, S. Condemi, A. Ronchitelli, K. Harvati, G. W. Weber, Early dispersal of modern humans in Europe and implications for Neanderthal behaviour. *Nature* **479**, 525–528 (2011).

72. J. Zilhão, W. E. Banks, F. D'Errico, P. Gioia, Analysis of site formation and assemblage integrity does not support attribution of the Uluzzian to modern humans at Grotta del Cavallo. *PLOS ONE* **10**, e0131181 (2015).

73. S. Faurby, M. Davis, R. Pedersen, S. D. Schowanek, A. Antonelli, J. C. Svenning, PHYLACINE 1.2: The phylogenetic atlas of mammal macroecology. *Ecology* **99**, 2626 (2018).

74. G. Terlato, V. Lubrano, M. Romandini, A. B. Marín-Arroyo, S. Benazzi, M. Peresani, Late Neanderthal subsistence at San Bernardino Cave (Berici Hills—Northeastern Italy) inferred from zooarchaeological data. *Alp. Mediterr. Quat.* **34**, 213–235 (2021).

75. W. Rendu, S. Costamagno, L. Meignen, M. C. Soulier, Monospecific faunal spectra in Mousterian contexts: Implications for social behavior. *Quat. Int.* **247**, 50–58 (2012).

76. G. A. Clark, C. Michael Barton, L. G. Straus, Landscapes, climate change & forager mobility in the Upper Paleolithic of northern Spain. *Quat. Int.* **515**, 176–187 (2019).

77. T. J. Heaton, P. Köhler, M. Butzin, E. Bard, R. W. Reimer, W. E. N. Austin, C. Bronk Ramsey, P. M. Grootes, K. A. Hughen, B. Kromer, P. J. Reimer, J. Adkins, A. Burke, M. S. Cook, J. Olsen, L. C. Skinner, Marine20—The marine radiocarbon age calibration curve (0–55,000 cal BP). *Radiocarbon.* **62**, 779–820 (2020).

78. M. Cortés-Sánchez, F. J. Jiménez-Espejo, M. D. Simón-Vallejo, C. Stringer, M. C. Lozano Francisco, A. García-Alix, J. L. Vera Peláez, C. P. Odriozola, J. A. Riquelme-Cantal, R. Parrilla Giráldez, A. Maestro González, N. Ohkouchi, A. Morales-Muñiz, An early Aurignacian arrival in southwestern Europe. *Nat. Ecol. Evol.* **3**, 207–212 (2019).

79. S. Talamo, V. Aldeias, P. Goldberg, L. Chiotti, H. L. Dibble, G. Guérin, J. J. Hublin, S. Madelaine, R. Maria, D. Sandgathe, T. E. Steele, A. Turq, S. J. P. Mcpherron, The new ^{14}C chronology for the Palaeolithic site of La Ferrassie, France: The disappearance of Neanderthals and the arrival of *Homo sapiens* in France. *J. Quat. Sci.* **35**, 961–973 (2020).

80. T. Higham, F. Brock, M. Peresani, A. Broglio, R. Wood, K. Douka, Problems with radiocarbon dating the Middle to Upper Palaeolithic transition in Italy. *Quat. Sci. Rev.* **28**, 1257–1267 (2009).

81. W. E. Banks, P. Bertran, S. Ducasse, L. Klaric, P. Lanos, C. Renard, M. Mesa, An application of hierarchical Bayesian modeling to better constrain the chronologies of Upper Paleolithic archaeological cultures in France between ca. 32,000–21,000 calibrated years before present. *Quat. Sci. Rev.* **220**, 188–214 (2019).

82. L. Philippe, P. Anne, Event date model: A robust Bayesian tool for chronology building. *Commun. Stat. Appl. Methods.* **25**, 131–157 (2018).

83. M. R. Bebber, A. J. M. Key, Optimal linear estimation (OLE) modeling supports Early Holocene (9000–8000 RCYBP) copper tool production in North America. *Am. Antiq.* **87**, 267–283 (2022).

84. A. J. M. Key, I. Jarić, D. L. Roberts, Modelling the end of the Acheulean at global and continental levels suggests widespread persistence into the Middle Palaeolithic. *Humanit. Soc. Sci. Commun.* **8**, 55 (2021).

85. D. A. Contreras, J. Meadows, Summed radiocarbon calibrations as a population proxy: A critical evaluation using a realistic simulation approach. *J. Archaeol. Sci.* **52**, 591–608 (2014).

86. O. García Puchol, A. Diez Castillo, S. Pardo-Gordó, New insights into the neolithisation process in southwest Europe according to spatial density analysis from calibrated radiocarbon dates. *Archaeol. Anthropol. Sci.* **10**, 1807–1820 (2018).

87. A. E. Thompson, J. P. Walden, A. S. Z. Chase, S. R. Hutson, D. B. Marken, B. Cap, E. C. Fries, M. R. G. Piedrasanta, T. S. Hare, S. W. Horn, G. J. Micheletti, S. M. Montgomery, J. Munson, H. Richards-Rissetto, K. Shaw-Müller, T. Ardren, J. J. Awe, M. K. Brown, M. Callaghan, C. E. Ebert, A. Ford, R. A. Guerra, J. A. Hoggarth, B. Kovacevich, J. M. Morris, H. Moyes, T. G. Powis, J. Yaeger, B. A. Houk, K. M. Prufer, A. F. Chase, D. Z. Chase, Ancient Lowland Maya neighborhoods: Average nearest neighbor analysis and kernel density models, environments, and urban scale. *PLOS ONE* **17**, e0275916 (2022).

88. A. Bonnier, M. Finné, E. Weiberg, Examining land-use through GIS-based kernel density estimation: A re-evaluation of legacy data from the Berbati-Limnes survey. *J. Field Archaeol.* **44**, 70–83 (2019).

89. M. J. Hamilton, J. Lobo, E. Rupley, H. Youn, G. B. West, The ecological and evolutionary energetics of hunter-gatherer residential mobility. *Evol. Anthropol.* **25**, 124–132 (2016).

90. E. Armstrong, P. O. Hopcroft, P. J. Valdes, A simulated Northern Hemisphere terrestrial climate dataset for the past 60,000 years. *Sci. Data* **6**, 265 (2019).

91. P. J. Valdes, E. Armstrong, M. P. S. Badger, C. D. Bradshaw, F. Bragg, M. Crucifix, T. Davies-Barnard, J. Day, A. Farnsworth, C. Gordon, P. O. Hopcroft, A. T. Kennedy, N. S. Lord, D. J. Lunt, A. Marzocchi, L. M. Parry, V. Pope, W. H. G. Roberts, E. J. Stone, G. J. L. Tourte, J. H. T. Williams, The BRIDGE HadCM3 family of climate models: HadCM3@Bristol v1.0. *Geosci. Model Dev.* **10**, 3715–3743 (2017).

92. B. Smith, I. C. Prentice, M. T. Sykes, Representation of vegetation dynamics in the modelling of terrestrial ecosystems: Comparing two contrasting approaches within European climate space. *Glob. Ecol. Biogeogr.* **10**, 621–637 (2001).

93. J. R. M. Allen, M. Forrest, T. Hickler, J. S. Singarayer, P. J. Valdes, B. Huntley, Global vegetation patterns of the past 140,000 years. *J. Biogeogr.* **47**, 2073–2090 (2020).

94. B. Smith, D. Wärlind, A. Arneth, T. Hickler, P. Leadley, J. Siltberg, S. Zaehle, Implications of incorporating N cycling and N limitations on primary production in an individual-based dynamic vegetation model. *Biogeosciences.* **11**, 2027–2054 (2014).

95. D. Lüthi, M. Le Floch, B. Bereiter, T. Blunier, J. M. Barnola, U. Siegenthaler, D. Raynaud, J. Jouzel, H. Fischer, K. Kawamura, T. F. Stocker, High-resolution carbon dioxide concentration record 650,000-800,000 years before present. *Nature* **453**, 379–382 (2008).

96. L. Zobler, A World Soil File for Global Climate Modelling. NASA Technical Memorandum 87802 (New York, 1986); https://daac.ornl.gov/SOILS/guides/ZoblerSoil1.html.

97. J. F. Hoffecker, Neanderthals and modern humans in Eastern Europe. *Evol. Anthropol.* **7**, 129–141. (1999).

98. R. Ø. Pedersen, S. Faurby, J.-C. Svenning, Extinctions have strongly reduced the mammalian consumption of primary productivity. *bioRxiv*, 2020.10.15.341297 [**Preprint**] (2020).

99. G. Rodríguez-Gómez, J. Rodríguez, J. A. Martín-González, A. Mateos, Evaluating the impact of *Homo*-carnivore competition in European human settlements during the early to middle Pleistocene. *Quatern. Res.* **88**, 129–151 (2017).

100. J. Zhang, B. Li, Y. Chen, M. Chen, T. Fang, Y. Liu, Eigenvector spatial filtering regression modeling of ground $PM_{2.5}$ concentrations using remotely sensed data. *Int. J. Environ. Res. Public Health* **15**, 1228 (2018).

101. P. Montero, J. A. Vilar, TSclust: An R package for time series clustering. *J. Stat. Softw.* **62**, 1–43 (2014).

102. S. Pederzani, K. Britton, V. Aldeias, N. Bourgon, H. Fewlass, T. Lauer, S. P. McPherron, Z. Rezek, N. Sirakov, G. M. Smith, R. Spasov, N. H. Tran, T. Tsanova, J. J. Hublin, Subarctic climate for the earliest Homo sapiens in Europe. *Sci. Adv.* **7**, eabi4642 (2021).

103. L. Marks, M. Makos, M. Szymanek, B. Woronko, J. Dzierżek, A. Majecka, Late Pleistocene climate of Poland in the mid-European context. *Quat. Int.* **504**, 24–39 (2019).

104. L. Krauß, A. Kappenberg, J. Zens, M. Kehl, P. Schulte, C. Zeeden, E. Eckmeier, F. Lehmkuhl, Reconstruction of Late Pleistocene paleoenvironments in southern Germany using two high-resolution loess-paleosol records. *Palaeogeogr. Palaeoclimatol. Palaeoecol.* **509**, 58–76 (2018).

105. A. Álvarez-Vena, D. J. Álvarez-Lao, C. Laplana, J. M. Quesada, J. Rojo, E. García-Sánchez, M. Menéndez, Environmental context for the Late Pleistocene (MIS 3) transition from Neanderthals to early modern humans: Analysis of small mammals from La Güelga Cave, Asturias, northern Spain. *Palaeogeogr. Palaeoclimatol. Palaeoecol.* **562**, 110096 (2021).

106. M. Fernández-García, M. Vidal-Cordasco, J. R. Jones, A. B. Marín-Arroyo, Reassessing palaeoenvironmental conditions during the Middle to Upper Palaeolithic transition in the Cantabrian region (Southwestern Europe). *Quat. Sci. Rev.* **301**, 107928 (2023).

107. A. Key, D. Roberts, I. Jarić, Reconstructing the full temporal range of archaeological phenomena from sparse data. *J. Archaeol. Sci.* **135**, 105479 (2021).

108. C. F. Clements, N. T. Worsfold, P. H. Warren, B. Collen, N. Clark, T. M. Blackburn, O. L. Petchey, Experimentally testing the accuracy of an extinction estimator: Solow's optimal linear estimation model. *J. Anim. Ecol.* **82**, 345–354 (2013).

109. R. M. Beyer, M. Krapp, A. Manica, High-resolution terrestrial climate, bioclimate and vegetation for the last 120,000 years. *Sci. Data* **7**, 236 (2020).

110. M. Krapp, R. M. Beyer, S. L. Edmundson, P. J. Valdes, A. Manica, A statistics-based reconstruction of high-resolution global terrestrial climate for the last 800,000 years. *Sci. Data* **8**, 228 (2021).

111. R. M. Beyer, M. Krapp, A. Eriksson, A. Manica, Climatic windows for human migration out of Africa in the past 300,000 years. *Nat. Commun.* **12**, 4889 (2021).

112. R. Beyer, M. Krapp, A. Manica, An empirical evaluation of bias correction methods for palaeoclimate simulations. *Clim. Past.* **16**, 1493–1508 (2020).

113. I. Harris, T. J. Osborn, P. Jones, D. Lister, Version 4 of the CRU TS monthly high-resolution gridded multivariate climate dataset. *Sci. Data* **7**, 109 (2020).

114. O. Peyron, M. Magny, S. Goring, S. Joannin, J. L. De Beaulieu, E. Brugiapaglia, L. Sadori, G. Garfi, K. Kouli, C. Ioakim, N. Combourieu-Nebout, Contrasting patterns of climatic changes during the Holocene across the Italian Peninsula reconstructed from pollen data. *Clim. Past.* **9**, 1233–1252 (2013).

115. B. A. S. Davis, M. Chevalier, P. Sommer, V. A. Carter, W. Finsinger, A. Mauri, L. N. Phelps, M. Zanon, R. Abegglen, C. M. Åkesson, F. Alba-Sánchez, R. Scott Anderson, T. G. Antipina, J. R. Atanassova, R. Beer, N. I. Belyanina, T. A. Blyakharchuk, O. K. Borisova, E. Bozilova, G. Bukreeva, M. Jane Bunting, E. Clò, D. Colombaroli, N. Combourieu-Nebout, S. Desprat, F. Di Rita, M. Djamali, K. J. Edwards, P. L. Fall, A. Feurdean, W. Fletcher, A. Florenzano, G. Furlanetto, E. Gaceur, A. T. Galimov, M. Gałka, I. García-Moreiras, T. Giesecke, R. Grindean, M. A. Guido, I. G. Gvozdeva, U. Herzschuh, K. L. Hjelle, S. Ivanov, S. Jahns, V. Jankovska, G. Jiménez-Moreno, M. Karpińska-Kołaczek, I. Kitaba, P. Kołaczek, E. G. Lapteva, M. Latałowa, V. Lebreton, S. Leroy, M. Leydet, D. A. Lopatina, J. A. López-Sáez, A. F. Lotter, D. Magri, E. Marinova, I. Matthias, A. Mavridou, A. M. Mercuri, J. M. Mesa-Fernández, Y. A. Mikishin, K. Milecka, C. Montanari, C. Morales-Molino, A. Mrotzek, C. M. Sobrino, O. D. Naidina, T. Nakagawa, A. B. Nielsen, E. Y. Novenko, S. Panajiotidis, N. K. Panova, M. Papadopoulou, H. S. Pardoe, A. Pędziszewska, T. I.

Petrenko, M. J. Ramos-Román, C. Ravazzi, M. Rösch, N. Ryabogina, S. S. Ruiz, J. Sakari Salonen, T. V. Sapelko, J. E. Schofield, H. Seppä, L. Shumilovskikh, N. Stivrins, P. Stojakowits, H. S. Svitavska, J. Święta-Musznicka, I. Tantau, W. Tinner, K. Tobolski, S. Tonkov, M. Tsakiridou, V. Valsecchi, O. G. Zanina, M. Zimny, The Eurasian Modern Pollen Database (EMPD), version 2. *Earth Syst. Sci. Data* **12**, 2423–2445 (2020).

116. M. Chevalier, B. A. S. Davis, O. Heiri, H. Seppä, B. M. Chase, K. Gajewski, T. Lacourse, R. J. Telford, W. Finsinger, J. Guiot, N. Kühl, S. Y. Maezumi, J. R. Tipton, V. A. Carter, T. Brussel, L. N. Phelps, A. Dawson, M. Zanon, F. Vallé, C. Nolan, A. Mauri, A. de Vernal, K. Izumi, L. Holmström, J. Marsicek, S. Goring, P. S. Sommer, M. Chaput, D. Kupriyanov, Pollen-based climate reconstruction techniques for late Quaternary studies. *Earth Sci. Rev.* **210**, 103384 (2020).

117. J. Damuth, Population density and body size in mammals. *Nature* **290**, 699–700 (1981).

118. S. J. McNaughton, M. Oesterheld, D. A. Frank, K. J. Williams, Ecosystem-level patterns of primary productivity and herbivory in terrestrial habitats. *Nature* **341**, 142–144 (1989).

119. M. J. Coe, D. H. Cumming, J. Phillipson, Biomass and production of large African herbivores in relation to rainfall and primary production. *Oecologia* **22**, 341–354 (1976).

120. J. Cebrian, J. Lartigue, Patterns of herbivory and decomposition in aquatic and terrestrial ecosystems. *Ecol. Monogr.* **74**, 237–259 (2004).

121. J. Cebrian, Patterns in the fate of production in plant communities. *Am. Nat.* **154**, 449–468 (1999).

122. L. Santini, N. J. B. Isaac, G. F. Ficetola, TetraDENSITY: A database of population density estimates in terrestrial vertebrates. *Glob. Ecol. Biogeogr.* **27**, 787–791 (2018).

123. D. Murakami, Spatial regression modeling using the spmoran package: Boston housing price data examples. arXiv:1703.04467 [stat.OT] (2017), doi:10.48550/arxiv.1703.04467.
